# Evaluating the Adjuvant Therapeutic Effects of Probiotic Strains *Lactococcus cremoris* and *Lacticaseibacillus paracasei* on Canine Atopic Dermatitis and Their Impact on the Gut and Skin Microbiome

**DOI:** 10.3390/ani15213098

**Published:** 2025-10-24

**Authors:** Hsiao-Wen Huang, Ting-Chen Yeh, Jui-Chun Hsieh, Ching-Wen Tsai, Ya-Jane Lee, Ming-Ju Chen

**Affiliations:** 1 Department of Animal Science and Technology, National Taiwan University, No. 50, Ln. 155, Sec. 3, Keelung Rd., Taipei 106037, Taiwan; d05628007@ntu.edu.tw (H.-W.H.); r10626016@ntu.edu.tw (T.-C.Y.); jchsieh0913@gmail.com (J.-C.H.); r10626003@ntu.edu.tw (C.-W.T.); 2 Institute of Veterinary Clinical Science, School of Veterinary Medicine, National Taiwan University, No. 1, Sec. 4, Roosevelt Rd., Taipei 106328, Taiwan; yajanelee@ntu.edu.tw; 3 Department of Internal Medicine, National Taiwan University Veterinary Hospital, No. 153, Sec. 3, Keelung Rd., Taipei 106319, Taiwan; 4 Center for Biotechnology, National Taiwan University, No. 81, Changxing St., Taipei 106038, Taiwan

**Keywords:** probiotic, canine atopic dermatitis, skin microbiome, gut microbiome, gut–skin axis, allergy

## Abstract

This study investigated the therapeutic potential of a novel probiotic combination consisting of *Lactococcus cremoris* subsp. *cremoris* MP01 and *Lacticaseibacillus paracasei* subsp. *paracasei* MP02, collectively referred to as LCP, in the treatment of canine atopic dermatitis (CAD). In a 60-day open-label, single-arm trial involving eight dogs, notable clinical improvements were observed following daily LCP treatment, as evidenced by decreasing trends in Canine Atopic Dermatitis Extent and Severity Index and Pruritus Visual Analogue Scale scores, as well as a significant reduction in serum immunoglobulin E levels (*p* < 0.05). Microbiome and short-chain fatty acid (SCFA) analyses were subsequently conducted in a representative subset of six dogs to explore the effects of LCP on the fecal and skin microbial ecosystems. Concomitant alterations in gut and skin microbiome were observed, including a significant reduction in abundance of *Erysipelotrichaceae* (*p* < 0.05) and non-significant decreasing trends in *Romboutsia*, *Escherichia*/*Shigella* spp., and *Shigella flexneri*, along with a trend toward increased SCFA production. Functional prediction using PICRUSt suggested potential involvement of immune- and infection-related signaling pathways, including those associated with nucleotide-binding oligomerization domain-like receptors, retinoic acid-inducible gene I-like receptors and Shigellosis, supporting the hypothesis that LCP may exert its effects through modulation of the gut–skin axis. These findings support LCP as a safe and promising adjunct therapy for CAD, offering a novel microbiome-targeted approach targeting both clinical symptoms and underlying dysbiosis. Further investigation is warranted to optimize probiotic formulations and better understand the mechanisms underlying microbiome-mediated immune modulation in canine allergy.

## Simple Summary

Atopic dermatitis is a common and distressing skin disease in dogs, often causing itching, discomfort, and reduced quality of life. Current treatments can help but are not always sufficient, and long-term use of medications may have side effects. This study explored whether a special combination of two beneficial probiotics, used as a daily supplement, could support dogs with this condition. Eight dogs with atopic dermatitis were given the probiotic blend for 60 days. Over this period, the dogs showed visible improvements, with reduced skin inflammation, less itching, and lower blood markers of allergy. To better understand how the supplement might work, the bacteria living in the gut and on the skin were also studied. The probiotic blend helped restore a healthier balance of microbes and suggested a possible role in strengthening the immune system. These findings indicate that probiotics may serve as a safe and helpful addition to standard treatments for dogs with atopic dermatitis. This approach could open new opportunities to improve the health and well-being of companion animals through nutrition-based strategies.

## 1. Introduction

Canine atopic dermatitis (CAD) is a prevalent, chronic allergic skin condition frequently encountered in clinical practice, characterized by persistent pruritus and scratching. The recent definition presented in the “Introduction to the International Consortium on Allergic Diseases in Animals (ICADA) 2023 CAD Pathogenesis Review Articles” advances our understanding of CAD as follows: “CAD is a hereditary, typically pruritic, and predominantly T-cell driven inflammatory skin disease involving an interplay between skin barrier abnormalities, allergen sensitization, and microbial dysbiosis [1]”. It is marked by pruritus, inflammation, and recurrent skin infections, affecting common sites such as the paws, face, ears, and abdomen. CAD is recognized as the second most common form of allergic dermatitis in dogs, following flea allergy dermatitis, with an estimated global incidence of approximately 10–15% [2]. Although current treatments primarily alleviate symptoms, CAD significantly impacts the quality of life of both affected dogs and their owners [3,4].

CAD manifests as a type I hypersensitivity reaction, predominantly driven by an imbalance between type 1 T helper (Th1) cells and type 2 T helper (Th2) cells. Upon antigen exposure, Th2 cells secrete cytokines, including interleukin (IL)-4, IL-5, IL-13, and IL-31, leading to the activation of B cells and the subsequent release of immunoglobulin E (IgE). Re-exposure to allergens triggers the release of IL-3, IL-4, IL-5, and various pro-inflammatory mediators, such as histamine and prostaglandins, resulting in increased vascular permeability and classic inflammatory responses, which include erythema, swelling, heat, and pain [5,6,7]. In dogs with atopic dermatitis, structural and biochemical alterations of the epidermal barrier play a pivotal role in disease development. Defects in the expression of structural proteins such as filaggrin [8,9], together with reduced levels of ceramides and free fatty acids, important lipid components of the stratum corneum, further impair epidermal integrity [10]. These changes result in increased transepidermal water loss (TEWL) and a weakened barrier function [11], allowing easier penetration of allergens, haptens, and irritants into the skin. This repetitive scratching disrupts the already compromised epidermal barrier, facilitating tissue fluid exudation. Moreover, the resulting pruritus often leads to excessive behaviors such as scratching, chewing, biting, and licking, which further aggravate barrier dysfunction, enhance allergen penetration, and promote colonization by opportunistic pathogens, including commensal bacteria such as *Staphylococcus pseudintermedius*, which can adhere more readily to damaged skin and exacerbate inflammation [12,13,14].

The skin and gut share numerous functions, including a dense network of nerves and blood vessels, and both play critical roles in the immune and endocrine systems while serving as barriers to the external environment [15,16]. Research has established a link between skin diseases and alterations in gut microbiota. Human patients with atopic dermatitis typically exhibit reduced fecal microbial diversity, along with diminished populations of beneficial bacteria known to produce short-chain fatty acids (SCFAs), such as *Lactobacillus*, *Bifidobacterium*, *Akkermansia*, *Faecalibacterium*, and *Coprococcus*. These changes are often accompanied by an increased abundance of potentially pathogenic species, including *Escherichia coli*, *Clostridium difficile*, *Staphylococcus aureus*, *Candida*, and *Rhodotorula* [17,18,19]. Although studies investigating gut microbiota in canine atopic dermatitis (CAD) are still limited and growing, similar patterns have been observed, with a lower abundance of *Faecalibacterium* [20] and an enrichment of *Escherichia-Shigella* [21]. This suggests a significant correlation between gut health and skin integrity, highlighting the mutual influence of dysbiosis between gut and skin microbiota and immune dysregulation [22].

Extensive studies have indicated that probiotics can modulate gut microbiota and maintain the integrity of the gut epithelium, thereby influencing overall immune balance by stimulating Th1 cells or modulating cytokine levels in regulatory T cells (Treg), ultimately improving allergic manifestations [23,24,25]. A recent study further demonstrated that heat-killed *Lactobacillus rhamnosus* and *Lactobacillus reuteri* stimulated canine peripheral blood mononuclear cells to produce a cytokine profile typically associated with an antiallergic response [26]. Despite these findings, clinical trials investigating the efficacy of probiotics in ameliorating CAD through the analysis of both gut and skin microbiota remain scarce. In prior investigations, our laboratory utilized an atopic dermatitis mouse model to demonstrate that a combination of *Lactococcus cremoris* subsp. *cremoris* MP01 and *Lacticaseibacillus paracasei* subsp. *paracasei* MP02 effectively reduced the Th1 to Th2 cells ratio in the spleen and decreased serum IgE levels, yielding positive effects on skin inflammation, epidermal proliferation, and skin integrity [27]. Therefore, this study aims to evaluate the efficacy of this mixed strain combination in improving clinical outcomes in CAD and to integrate gut and skin microbiome analyses to explore the impacts of probiotics on the pathophysiology of CAD.

## 2. Materials and Methods

### 2.1. Bacterial Strains

*Lc. cremoris* subsp. *cremoris* MP01 and *L. paracasei* subsp. *paracasei* MP02 were isolated from traditional fermented milk. The probiotic mixture was cultured and activated in Lactobacilli MRS broth (Neogen Corporation, Lansing, MI, USA) at 30 °C for 24 h.

### 2.2. Preparation of Probiotic Capsule

The dosage for dogs with atopic dermatitis (AD) was established based on a previous study [27]. The freeze-dried probiotic powder, containing *Lc. cremoris* subsp. *cremoris* MP01 and *L. paracasei* subsp. *paracasei* MP02, was produced by Grape King Bio, Ltd. (Taoyuan, Taiwan). These probiotic strains were mixed in a 2:1 ratio and encapsulated to form LCP capsules, each containing 3.1 × 10^10^ CFU.

### 2.3. Clinical Canine Trial

The open-label, single-arm trial was approved by the Institutional Animal Care and Use Committee of National Taiwan University (IACUC approval no: NTU-109-EL-000146). All owners provided written consent to participate in this study. Recruitment was conducted from August to November 2021.

The preliminary selection criteria were adapted from previous research [28,29]. There were no restrictions regarding the dogs’ age, weight, gender, breed, or sterilization status. However, dogs were excluded if they were pregnant, nursing, or had other health issues such as cancer. CAD was diagnosed clinically by exclusion, incorporating both owner-provided medical history and clinical assessment. Medical history included information on regular external parasite prevention, previous treatments for pruritus, and any recent antimicrobial or antifungal therapy. All enrolled dogs had not received any antibiotic or antifungal treatment for bacterial or fungal skin infections prior to study initiation. At enrollment, microscopic examinations (skin scrapings and scale evaluations from pruritic sites) were performed to rule out external parasitic and fungal infections, and no dogs presented with visible purulent skin lesions. Additionally, all dogs were required to have been on a stable diet, consuming the same commercial dog food without any supplements or treats for at least eight weeks prior to enrollment. Dogs displaying any signs suggestive of food-related allergic reactions or those with recent dietary changes were excluded to avoid confounding effects. Throughout the study period, all enrolled dogs continued their original diet to ensure consistency and minimize dietary influences on study outcomes.

Eligible dogs needed to exhibit itching and erythema on their skin while meeting at least five of the following conditions:

(1) The initial onset of symptoms occurred at or before 3 years of age.
(2) The dog primarily resided indoors.
(3) Itching symptoms significantly improved with steroid treatment.
(4) Skin lesions were primarily located on the ear pinna, interdigital spaces, around the mouth, eyes, dorsal surfaces of joints, and inguinal areas.
(5) The edges of the ears appeared normal.
(6) The skin of the lower back was normal.

Further evaluations included a complete blood count (CBC) to assess parameters such as leukocyte and erythrocyte levels, confirming the absence of significant inflammatory responses, nutritional deficiencies, or leukopenia, and ensuring that each dog was in an appropriate condition for the trial. All enrolled dogs remained on stable, pre-existing AD treatment regimens throughout the study period, with no recent changes or therapies initiated within two weeks prior to enrollment.

Dogs meeting the eligibility criteria received one LCP capsule daily for 60 consecutive days. Clinical assessments and sample collections were performed on Days 0, 30, and 60. Clinical indices, including the Canine Atopic Dermatitis Extent and Severity Index (CADESI) and Pruritus Visual Analogue Scale (PVAS), as well as blood-based immune markers (total IgE, IL-4, and IFN-γ) were evaluated in all enrolled dogs. Fecal and skin samples for microbiota analysis were collected at baseline (Day 0) and at the end of the LCP treatment (Day 60). Fecal samples, collected as representatives of the gut microbiota, were also used for short-chain fatty acid (SCFA) analysis. Fecal samples were collected by the dog owners immediately after defecation using sterile tubes without preservative solution, stored at −18 °C at home, and transported to the laboratory under chilled conditions within 2 h. Upon arrival, samples were stored at −80 °C until further analysis. For skin microbiota sampling, cotton swabs were used to collect bacteria from four anatomical sites: around the mouth, ears, armpits, and groin. Swabs were pre-moistened with 0.85% sterile sodium chloride solution, used to swab the surface of each targeted site, and then returned to the same solution. Skin swab samples were stored at −20 °C until processing.

### 2.4. Assessment of the Degree of Atopic Dermatitis and Pruritus

To evaluate the severity level of atopic dermatitis, the CADESI-04 [30] and the Pruritus Visual Analog Scale (PVAS) [31] were used. For the CADESI-04 assessment, the levels of erythema, lichenification, excoriation, and alopecia were inspected and graded in 20 body areas on a scale from 0 (none) to 3 (severe), with a maximum total score of 180. The PVAS was completed by the dog owners to assess the degree of itching on a scale from 0 (not itchy) to 10 (extremely itchy).

### 2.5. Cytokine Production from Peripheral Blood Mononuclear Cells (PBMCs)

Two milliliters of blood were centrifuged at 3,300× *g* for 10 min, after which the plasma was collected and stored at −80 °C. The remaining cellular fraction was resuspended in Dulbecco’s phosphate-buffered saline (DPBS; Thermo Fisher Scientific Inc., Waltham, MA, USA) to a final volume of 4 mL. This suspension was carefully layered over 3 mL of Ficoll-Paque Premium (Cytiva, Marlborough, MA, USA) and centrifuged at 400× *g* for 35 min at 18 °C. The monocellular cells were collected and suspended in Roswell Park Memorial Institute (RPMI) 1640 medium (Sigma-Aldrich Inc., St. Louis, MO, USA) supplemented with heated-inactivated fetal bovine serum (FBS) and an antibiotic–antimycotic solution (Corning Inc., Corning, NY, USA). Cell viability was assessed using the trypan blue method. For the cytokine stimulation assays, viable PBMCs ranging from 5.2 × 10^5^ to 7.6 × 10^6^ cells per well were seeded and cultured in medium supplemented with concanavalin A (Sigma-Aldrich Inc., St. Louis, MO, USA) at 37 °C in a 5% CO_2_ atmosphere for 24 h. After stimulation, the culture was centrifuged at 400× *g* for 10 min at 4 °C to collect the supernatant for cytokine quantification. The remaining cell pellet was lysed for total protein analysis. Cytokine concentrations were normalized to the corresponding protein levels to reduce inter-well variability and improve the accuracy of comparative analyses.

Canine-specific enzyme-linked immunosorbent assay (ELISA) kits were used to quantify IL-4 (Bioassay Technology Laboratory, Shanghai, China) and interferon-gamma (IFN-γ; R&D Systems, Minneapolis, MN, USA) in culture supernatants collected from stimulated PBMCs. The Th1/Th2 balance was evaluated by calculating the ratio of IFN-γ to IL-4 concentrations. According to the manufacturers, the intra-assay CVs were <8% for IL-4 and <10% for IFN-γ, and the inter-assay CVs were <10% for IL-4 and <9% for IFN-γ.

### 2.6. IgE Production in Serum

Serum IgE concentrations were determined using a commercially available canine-specific enzyme-linked immunosorbent assay (ELISA) kit (LifeSpan BioSciences Inc., Newark, CA, USA), according to the manufacturer’s instructions. All serum samples and standards were run in duplicate to ensure accuracy and reproducibility. The assay’s reported performance characteristics include an intra-assay coefficient of variation (CV) of less than 8% and an inter-assay CV of less than 10%.

### 2.7. Analysis of Fecal and Skin Microbiota

Genomic DNA was extracted from fecal samples using a modified version of a previously described protocol [32]. Briefly, fecal material was homogenized in an extraction buffer with bead beating using the FastPrep-24™ 5G Instrument (MP Biomedicals, Irvine, CA, USA). DNA was then purified by phenol-chloroform extraction and precipitated with isopropanol. The resulting DNA was resuspended in TE buffer and stored at −20 °C until further analysis. For skin swab samples, the collection tubes were vortexed, and 0.75 to 1 mL of the suspension was transferred and centrifuged at 16,100× *g* for 10 min at 4 °C to pellet the bacteria. Genomic DNA was subsequently extracted from the pellets using the same protocol described above, following the addition of extraction buffer. DNA concentration was quantified with a Qubit 4.0 fluorometer (Thermo Fisher Scientific, Waltham, MA, USA) and adjusted to 1 ng/µL for subsequent experiments. Third-generation sequencing was conducted by BIOTOOLS Co., Ltd. (Taipei, Taiwan). Universal primers 27F (5′-AGRGTTYGATYMTGGCTCAG-3′) and 1492R (5′ RGYTACCTTGTTACGACTT-3′) were used to amplify the full length 16S rRNA gene covering the V1–V9 regions. SMRTbell library were constructed and sequenced using the PacBio Sequel IIe system (Menlo Park, CA, USA) in circular consensus sequencing (CCS) mode. High-fidelity (HiFi) reads with a quality score (RQ) greater than 30 were retained for downstream analysis. All fecal and skin microbiota samples were processed and sequenced in a single batch to minimize potential batch effects. These reads were then processed using the DADA2 package (v1.20) in R (v3.6.0) to denoise and generate amplicon sequence variants (ASVs), with each ASV defined as one species cluster [33,34]. The ASVs were classified into their taxonomy using QIIME2 (v2021.4; http://qiime2.org; accessed on 23 September 2022) and the NCBI 16S ribosomal RNA database (2021.1) [35,36,37,38,39,40].

In this study, α diversity was assessed using multiple indices: Shannon, which account for both richness and evenness of the community; observed features, which estimate microbial richness based on ASV counts; and Pielou’s evenness, which measures how uniformly species are distributed. β diversity was evaluated using Bray–Curtis dissimilarity to quantify differences in community composition between samples, and these dissimilarities were visualized through Principal Coordinates Analysis (PCoA) [41,42,43,44,45]. In addition, functional abundances inferred from 16S rRNA sequencing data were predicted using PICRUSt (Phylogenetic Investigation of Communities by Reconstruction of Unobserved States). The predicted functional genes were further annotated against the Kyoto Encyclopedia of Genes and Genomes (KEGG) database to examine potential changes in microbial pathways related to gut and skin microbiota [46].

### 2.8. Short-Chain Fatty Acids Analysis

Short-chain fatty acids (SCFAs), specifically acetate, propionate, and butyrate, were quantified from fecal samples following a previously established protocol with minor modifications [47]. Briefly, 0.2 g of feces was mixed with 0.8 mL of 70% ethanol and homogenized for 40 s using a FastPrep-24™ 5G Instrument (MP Biomedicals, Irvine, CA, USA). The mixture was then centrifuged at 16,100× *g* for 10 min at 4 °C. The resulting supernatant was collected for analysis. After a series of procedures, the extracted SCFAs were subsequently dissolved in 250 µL of methanol for quantification. A PU-2089 quaternary high-performance liquid chromatography (HPLC) pump (Jasco International Co. Ltd., Tokyo, Japan) equipped with a Reprosil 100 C18 (5 μM, 250 × 4.6 mm; Dr. Maisch GmbH, Ammerbuch, Germany) was used to quantify the concentration of fecal acetic acid, propionic acid, and butyric acid. The mobile phase consisted of acetonitrile, methanol, and ultrapure water at a volume ratio of 30, 16, and 54, respectively, and the pH was adjusted to 4.5 using trifluoroacetic acid. The flow rate was set at 1.1 mL/min, with a column temperature of 50 °C, and detection was performed at a wavelength of 400 nm.

### 2.9. Statistical Analysis

All data were presented as mean ± standard error of the mean (SEM). Longitudinal changes in clinical indices, including CADESI and PVAS scores, were analyzed using linear mixed-effects models (LMM), with time (day 0, day 30, day 60) as a fixed effect and dog ID as a random effect to account for repeated measurements within individuals. Estimated marginal means and corresponding 95% confidence intervals (CIs) were calculated. The Shapiro–Wilk test was used to assess normality. Depending on the distribution, either the paired *t*-test or the Wilcoxon signed-rank test was applied using GraphPad Prism v10.4.1 (GraphPad Software Inc., Boston, MA, USA) and SAS v9.4 (SAS Institute Inc., Cary, NC, USA). A *p*-value < 0.05 was considered statistically significant. Findings with *p*-values between 0.05 and 0.10 were interpreted as statistical trends, consistent with the exploratory nature of this pilot study, and were reported to highlight biologically suggestive patterns that may warrant further investigations. Given the limited sample size, no adjustments for multiple comparisons were applied. As microbiota data were expressed as relative abundances, non-parametric tests were used throughout. The Wilcoxon signed-rank test was applied for paired comparisons, and the Wilcoxon rank-sum test for unpaired comparisons. Spearman’s rank correlation analysis was used to examine associations between specific bacterial taxa and atopic dermatitis-related indicators, including CADESI scores and serum concentrations of total IgE, IL-4, and IFN-γ.

## 3. Results

### 3.1. Clinical and Immunological Responses to LCP Treatment in Dogs with Atopic Dermatitis

**Figure 1:**
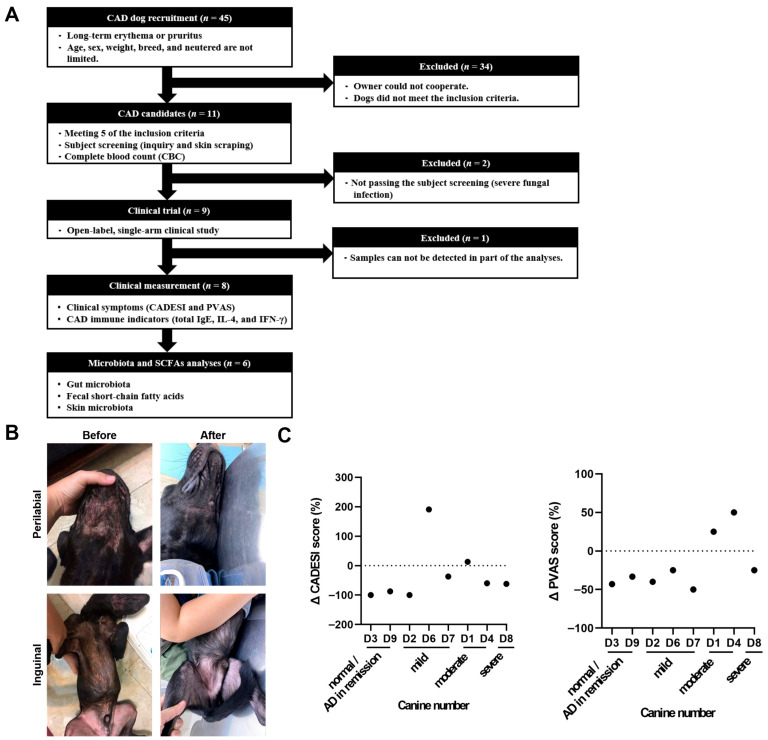
Study design and the effects of LCP treatment on symptoms in dogs with atopic dermatitis (AD). (**A**) Study design. (**B**) Schematic representation of skin lesions in the perilabial area (upper) and inguinal areas (lower) of the recruited dog before and after 60 days of LCP 60 days of LCP treatment. (**C**) Changes in CADESI and PVAS score. Δ (%) = 100 × (post-treatment score on day 60 − pretreatment score on day 0)/pretreatment score on day 0.

A total of 45 dogs were initially screened for eligibility in this study. Following veterinary assessment, 11 dogs met the inclusion criteria. Two dogs were excluded prior to study initiation due to unresolved fungal infections, and one additional dog was excluded from the analysis due to incomplete cytokine data, specifically the absence of IFN-γ measurements. Consequently, eight dogs completed the 60-day clinical trial and were included in the clinical assessments (CADESI and PVAS) and immune profiling (serum IgE, IL-4, and IFN-γ levels) (Figure 1A). The median body weight of the enrolled dogs was 10.4 kg, ranging from 2.7 to 32 kg, with a female-to-male ratio of 1:1. According to the AAHA Canine Life Stage Guidelines [48], two dogs were classified as young (1–3 years), three as adults (3–7 years), and three as seniors (over 8 years) (Supplementary Table S1).

Based on the CADESI scores assessed on day 0, dogs were classified according to the severity of their atopic dermatitis symptoms: two dogs were categorized as having normal or reduced AD symptoms (CADESI score < 10), three dogs were classified as mild (score = 10–34), two as moderate (score = 35–59), and one as severe (score ≥ 60) (Supplementary Table S2). After 60 days of LCP treatment, most dogs exhibited visible improvements in skin condition, including reduced erythema and alopecia (Figure 1B). The extent of improvement varied among individuals, as reflected in the percentage reduction in CADESI scores across dogs. Overall, CADESI and PVAS assessments indicated that up to 75% of the dogs showed decreases in severity scores (Figure 1C). However, linear mixed-effects model (LMM) analysis detected no statistically significant differences in CADESI or PVAS scores across the three time points, suggesting that although some dogs demonstrated individual clinical improvements, the overall changes were not statistically significant (Supplementary Table S3). Pairwise comparison results between different time points also showed consistent lack of statistical significance (Supplementary Figure S1).

**Figure 2:**
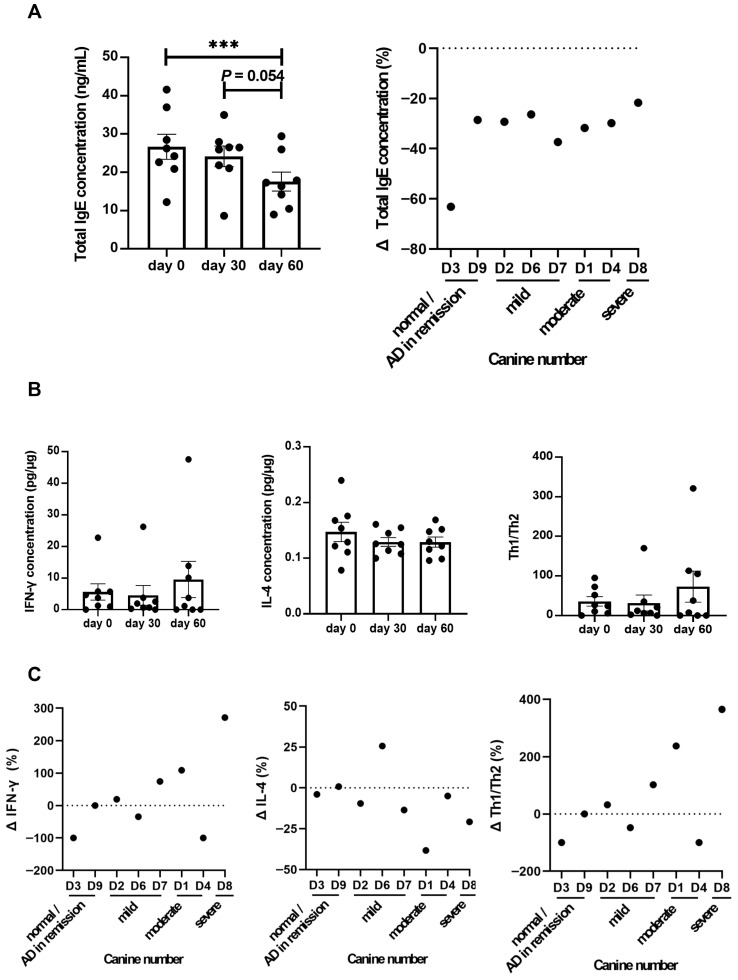
Effects of LCP treatment on IgE and cytokine levels in dogs with atopic dermatitis (AD). (**A**) Serum total IgE levels and changes in total IgE level after 60 days of LCP treatment. (**B**) IFN-γ, IL-4, and the Th1/Th2 cytokines ratio in PBMCs after 30 and 60 days of LCP treatment. (**C**) Changes in IFN-γ, IL-4, and the Th1/Th2 ratio. Data were presented as mean ± SEM (*n* = 8). Each dot represented an individual dog. Δ (%) = 100 × (post-treatment level on Day 60 − pretreatment level on Day 0)/pretreatment level on Day 0. The Th1/Th2 ratio was calculated as IFN-γ concentration (pg/μg)/IL-4 concentration (pg/μg). Cytokine levels were determined using canine-specific ELISA kits with validated performance: intra-assay CVs were <8% for IgE and IL-4, and <10% for IFN-γ; inter-assay CVs were <10% for IgE and IL-4, and <9% for IFN-γ. *** *p* < 0.001.

Total IgE levels, an important marker of atopic dermatitis, significantly decreased following LCP treatment (*p* < 0.05) (Figure 2A). Individual analyses showed a consistent reduction in total IgE levels across all dogs. While total IgE commonly reflects allergic activity, it is acknowledged that a subset of dogs with atopic-like dermatitis may not exhibit elevated IgE levels. In addition, IFN-γ levels showed an increasing trend and IL-4 levels a decreasing trend by day 60 compared to baseline, although these changes were not statistically significant (Figure 2B). Further individual analysis indicated that half of the eight dogs exhibited upregulation in Th1/Th2 levels (Figure 2C). Additionally, no adverse clinical signs or gastrointestinal symptoms were observed in any of the dogs during the 60-day LCP administration period. Veterinary examinations and owner-reported observations consistently indicated the absence of treatment-related side effects.

### 3.2. Fecal Microbiota Composition in Dogs with Atopic Dermatitis Before and After LCP Treatment

**Figure 3:**
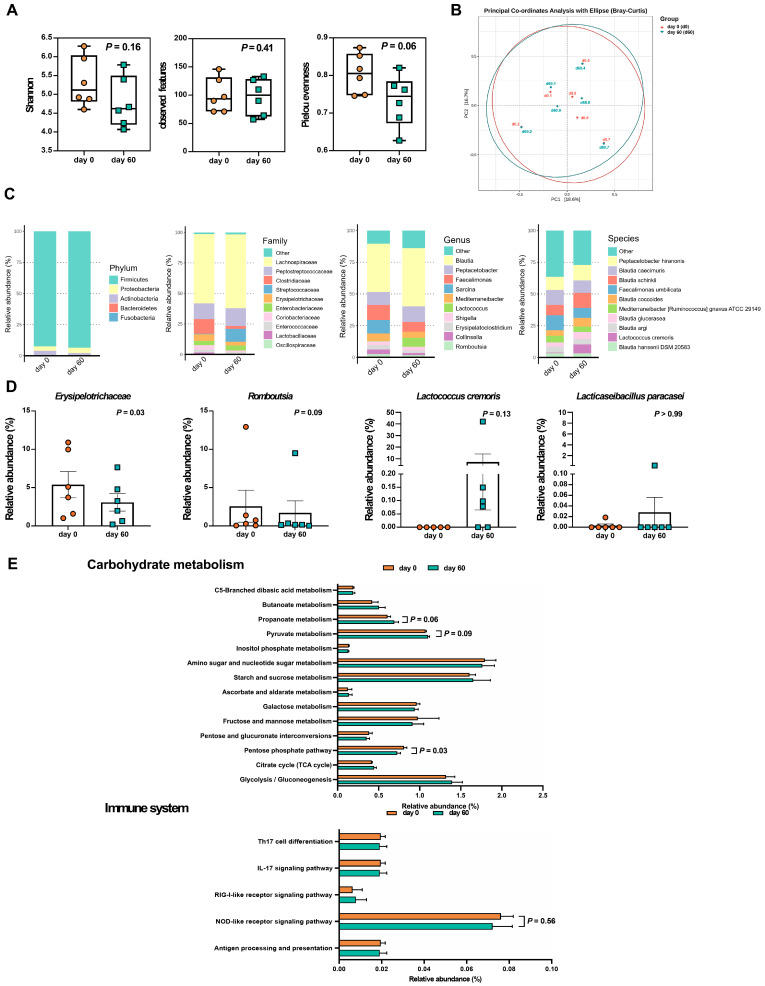
Effects of LCP treatment on fecal microbiota in dogs with atopic dermatitis (AD). (**A**) Alpha-diversity indices. (**B**) PCoA plot representing beta diversity (Bray–Curtis) among individual dogs with AD. (**C**) Relative abundances of the most dominant bacterial phyla, families, genera, and species. (**D**) Relative abundance of specific bacterial taxa. (**E**) Relative abundance of KEGG level 3 pathways related to carbohydrate metabolism and immune system functions before (day 0) and after 60 days of LCP treatment. Data were presented as mean ± SEM (*n* = 6). Each dot represents an individual dog. “Con” indicates samples before LCP treatment, while “Pro” represents samples after 60 days of LCP treatment.

To identify potential bacterial biomarkers, fecal microbiota analyses were performed on a representative subset of six dogs that completed the study and had complete paired fecal samples collected at both baseline (Day 0) and after 60 days of LCP treatment. Dogs were categorized into two groups based on CADESI severity: moderate to severe (D1, D4, D8) and normal/remission to mild (D2, D3, D6, D7, D9). All three dogs in the moderate to severe group were included to ensure adequate representation. Among the five dogs with mild symptoms, D3 was excluded due to a near-baseline CADESI score that showed minimal change post-treatment, and D6 was excluded due to a substantial increase in CADESI score (~200%) after LCP treatment (Figure 1C). This selection strategy aimed to reduce confounding variability and better associate microbial changes with clinical improvement. Skin microbiota were also analyzed for these six dogs, with detailed findings presented in the subsequent sections. To ensure robust microbiota profiling, sequencing depth was evaluated across all fecal samples. After quality filtering and chimera removal, the average number of non-chimeric reads per sample was 15,870, with a range of 13,227 to 17,398 reads. Sequencing depth was consistent across fecal samples, providing sufficient coverage for taxonomic profiling and diversity analysis. Figure 3 illustrates changes in the composition of fecal microbiota before and after the treatment, highlighting key taxonomic shifts following LCP treatment. Compared to Day 0, the Shannon index (*p* = 0.16) and observed features (*p* = 0.41) showed no significant changes, while Pielou’s evenness exhibited a trend toward reduction (*p* = 0.06) on Day 60 (Figure 3A). The PCoA plot representing beta diversity, evaluated using Bray–Curtis dissimilarity, showed that PC1 and PC2 accounted for 18.6% and 16.7% of the total variance, respectively (Figure 3B). Similar patterns in the PCoA plot before and after LCP treatment suggested minimal changes in the composition of core fecal bacteria. However, alterations were observed at different taxonomic levels.

In this study, fecal microbiota analysis of dogs with AD revealed five dominant phyla. Prior to LCP treatment, the relative abundances were as follows: *Firmicutes* (92.43%), *Proteobacteria* (3.53%), *Actinobacteria* (3.77%), *Bacteroidetes* (0.20%), and *Fusobacteria* (0.08%). After treatment, the composition shifted to *Firmicutes* (93.63%), *Proteobacteria* (4.15%), *Actinobacteria* (1.70%), *Bacteroidetes* (0.51%), and *Fusobacteria* was no longer detected (Figure 3C). However, none of these changes in phylum-level relative abundance reached statistical significance.

An in-depth analysis identified 25 bacterial families within the phylogenetic hierarchy. Among these, the top ten families, considered dominant, collectively accounted for approximately 98.51% of the total microbial community in both groups (Figure 3C). *Lachnospiraceae* was the most prevalent family, increasing from 56.70% before LCP treatment to 60.55% afterward. *Peptostreptococcaceae* ranked second, with relative abundance increasing from 13.04% to 14.64% post-treatment. Other families, listed by relative abundance, included *Clostridiaceae* (11.91% pre- vs. 2.27% post-treatment), *Streptococcaceae* (0.51% vs. 10.58%), *Erysipelotrichaceae* (5.38% vs. 3.08%), *Enterobacteriaceae* (3.53% vs. 4.15%), *Coriobacteriaceae* (3.62% vs. 1.65%), *Enterococcaceae* (2.43% vs. 0.53%), *Lactobacillaceae* (1.31% vs. 0.08%), and *Oscillospiraceae* (0.19% vs. 0.88%). Among these, only the decrease in *Erysipelotrichaceae* reached statistical significance (*p* = 0.03).

At the genus level, a total of 75 genera were identified, with the top ten accounting for 87.88% of the total average microbial composition (Figure 3C). *Blautia* was the most abundant genus, increasing from 38.07% before LCP treatment to 46.07% afterward. *Peptacetobacter* followed, rising from 10.22% to 12.09% with probiotic intake. Other notable genera included *Faecalimonas* (decreasing from 11.85% to 7.88%), *Sarcina* (dropping from 10.65% to undetectable levels), *Mediterraneibacter* (slightly decreasing from 5.88% to 4.75%), and *Lactococcus* (increasing from 0.39% to 7.06%). Meanwhile, *Shigella* remained unchanged at 2.91%, while *Erysipelatoclostridium* (3.44% to 2.02%), *Collinsella* (3.62% to 1.65%), and *Romboutsia* (2.57% to 1.71%) showed slight reductions. Among these genera, *Romboutsia* demonstrated a trend toward decreased abundance following LCP treatment (*p* = 0.09).

Overall, 137 bacterial species were identified, with the top ten species collectively accounting for 68.12% of the total average microbial composition. *Peptacetobacter hiranonis* was the most abundant species, increasing from 10.22% before LCP treatment to 12.09% afterward. *Blautia caecimuris* followed, with relative abundances of 11.78% before and 9.85% after probiotic intake. Additional species in descending order of relative abundance, included *Blautia schinkii* (8.16% pre- and 11.83% post-probiotic), *Faecalimonas umbilicata* (11.85% pre- and 7.88% post-probiotic), *Blautia coccoides* (4.30% pre- and 6.72% post-probiotic), *Mediterraneibacter* [*Ruminococcus*] *gnavus* ATCC 29149 (5.22% pre- and 4.35% post-probiotic), *Blautia glucerasea* (3.40% pre- and 5.61% post-probiotic), *Blautia argi* (4.67% pre- and 4.28% post-probiotic), *Lactococcus cremoris* (0.38% pre- and 7.06% post-probiotic), and *Blautia hansenii* DSM 20583 (3.42% pre- and 3.15% post-probiotic). However, none of these species-level changes in relative abundance reached statistical significance.

We employed the Wilcoxon signed-rank test to assess changes in bacterial relative abundance before and after treatment at various taxonomic levels (Figure 3D). The results revealed a significant decrease in *Erysipelotrichaceae* at the family level (*p* < 0.05) and a decreasing trend in *Romboutsia* at the genus level (*p* = 0.09). Additionally, *Lactococcus cremoris*, one of the strains included in the LCP formulation, was detected in fecal samples from four out of six dogs on Day 60, compared to only one dog at baseline. This suggests increased detectability of the administered strain following LCP treatment, reflecting its transient presence and potential gut passage during the intervention period. For additional reference, Supplementary Tables S4–S7 presented the median (range) of relative abundance for dominant fecal bacterial phyla, families, genera, and species, along with *p*-values and FDR-adjusted q-values. To further illustrate inter-individual variability in fecal microbiota composition, stacked bar plots showing the relative abundance of bacterial taxa at the phylum, family, genus, and species levels for each dog were provided in Supplementary Figure S2.

### 3.3. Predicted Fecal Microbial Functions and SCFA Profiles After LCP Treatment

To investigate the potential functional consequences of fecal microbiota changes, and how these changes in the gut environment might influence skin symptoms, functional predictions were generated using PICRUSt based on KEGG orthologs inferred from 16S rRNA data. These predictions focused on carbohydrate metabolism and immune system-associated inflammation (Figure 3E). A significant decrease (*p* < 0.05) was observed in the predicted abundance of KEGG pathway genes involved in the pentose phosphate pathway (ko00030) in the LCP-treated group. Additionally, there was a trend toward higher predicted abundances of genes involved in pyruvate metabolism (ko00620) and propanoate metabolism (ko00640) following LCP treatment. A slight increase in predicted genes related to butanoate metabolism (ko00650) was also observed (*p* = 0.22), which may be relevant to SCFA production. However, no statistically significant changes were detected in immune system-related pathways based on functional prediction (Supplementary Table S8).

**Figure 4:**
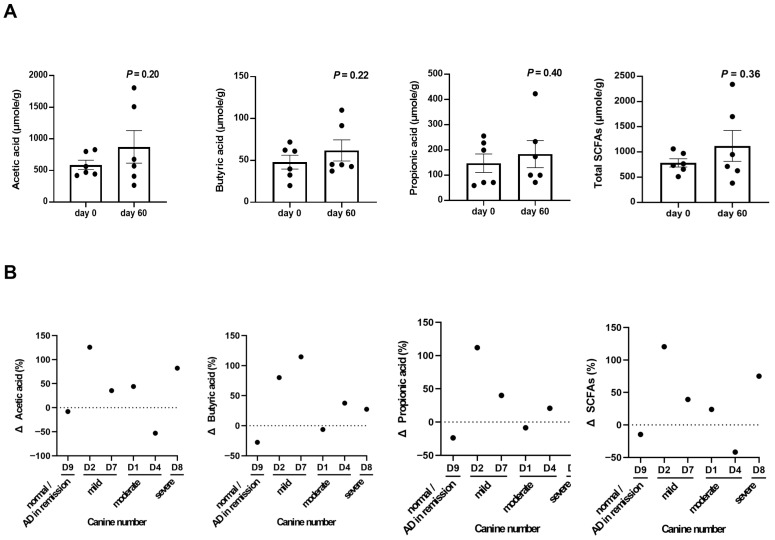
Effects of LCP treatment on fecal short-chain fatty acid (SCFA) levels in dogs with atopic dermatitis (AD). (**A**) SCFA concentrations before (day 0) and after 60 days of LCP treatment. (**B**) Changes in SCFA levels after 60 days of LCP treatment. Data were presented as mean ± SEM (*n* = 6). Each dot represents an individual dog. Δ (%) = 100% × (post-treatment level on day 60 − pretreatment level on day 0)/pretreatment level on day 0.

To further explore the functional consequences of fecal microbiota modulation, short-chain fatty acid (SCFA) levels were measured in fecal samples from the same six dogs used in microbiota analysis. SCFA quantification was performed at baseline and after 60 days of LCP treatment to evaluate potential gut-derived metabolic changes contributing to skin health. As shown in Figure 4, although no statistically significant difference were observed before and after LCP treatment, increased levels of acetic acid, propionic acid, and butyric acid were detected in the feces of 4 out of 6 tested dogs after 60 days of LCP treatment.

### 3.4. Skin Microbiota Composition in Dogs with Atopic Dermatitis Before and After LCP Treatment

**Figure 5:**
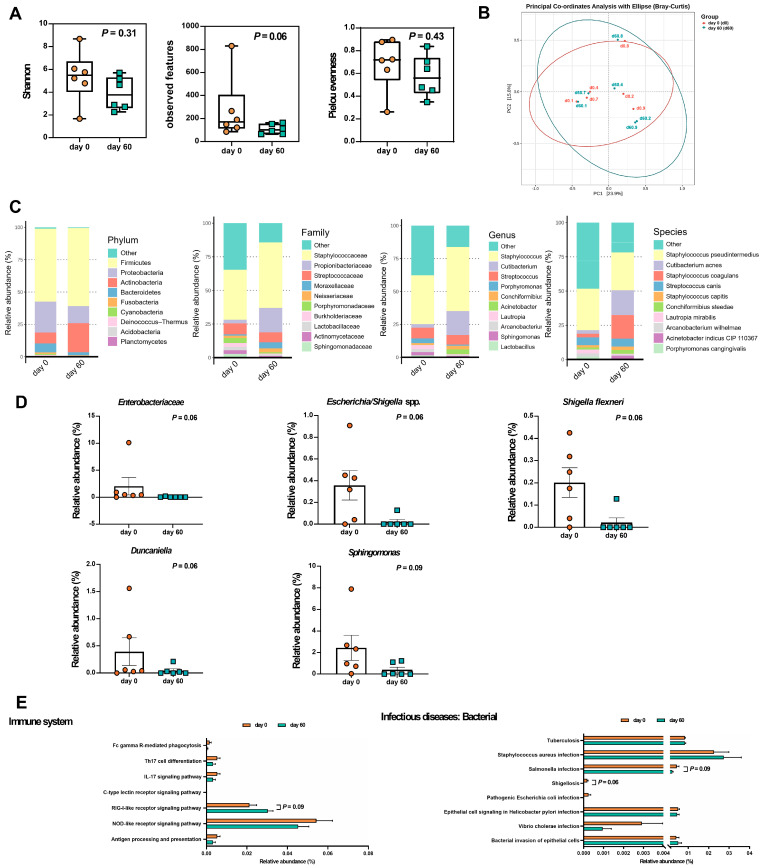
Effects of LCP treatment on skin microbiota in dogs with atopic dermatitis (AD). (**A**) Alpha-diversity indices. (**B**) PCoA plot representing beta diversity (Bray–Curtis) among individual dogs with AD. (**C**) Relative abundances of the most dominant bacterial phyla, families, genera, and species. (**D**) Relative abundance of specific bacterial taxa. (**E**) Relative abundances of KEGG level 3 pathways related to immune system functions and bacterial infectious diseases before (day 0) and after 60 days of LCP treatment. Data were presented as mean ± SEM (*n* = 6). Each dot represents an individual dog. “Con” indicates samples before LCP treatment, while “Pro” represents samples after 60 days of LCP treatment.

To investigate the effects of LCP treatment on the skin microbiota, we analyzed bacterial composition changes in skin samples collected from the same six dogs used in the gut microbiota analysis, with paired samples obtained at baseline and Day 60. Given the low-biomass nature of skin microbiota, sequencing depth was carefully assessed. After quality filtering and chimera removal, the average number of non-chimeric reads per skin sample was 12,042 (range: 9,376–20,421), which was sufficient for taxonomic characterization across all samples. The alpha diversity of the skin microbiota in dogs with AD is illustrated in Figure 5A. The richness index showed a decreasing trend after 60 days of LCP treatment (*p* = 0.06). In the PCoA plot, beta diversity exhibited a shift, with PC1 and PC2 accounting for 23.9% and 15.6% of the total skin microbiota composition, respectively (Figure 5B).

The composition of the skin microbiota in dogs with AD is illustrated in Figure 5C. Before LCP treatment, the predominant phyla in the tested dogs’ skin microbiota were *Firmicutes* (56.36%), *Proteobacteria* (23.83%), *Actinobacteria* (8.52%), *Bacteroidetes* (6.88%), *Fusobacteria* (1.11%), *Cyanobacteria* (0.93%), *Deinococcus*-*Thermus* (0.74%), *Acidobacteria* (0.36%), and *Planctomycetes* (0.21%). After treatment, the composition shifted to *Firmicutes* (60.25%), *Proteobacteria* (13.34%), *Actinobacteria* (22.35%), *Bacteroidetes* (2.42%), *Fusobacteria* (0.31%), *Cyanobacteria* (0.15%), *Deinococcus*-*Thermus* (0.28%), *Acidobacteria* (0.37%), and *Planctomycetes* (0.03%). Among these, *Planctomycetes* showed a trend toward decreased abundance following LCP treatment (*p* = 0.06).

Among the 178 identified families, the top ten collectively accounted for 75.59% of the total microbiota composition in both groups. *Staphylococcaceae* was the most prevalent, increasing from 37.20% before LCP treatment to 48.71% afterward. Other notable families included *Propionibacteriaceae* (increasing from 2.82% to 18.20%) and *Streptococcaceae* (slightly decreasing from 8.06% to 7.31%). However, none of these changes in relative abundance reached statistical significance. At the genus level, 411 genera were identified, with the top ten genera representing 73.09% of the overall skin microbiota. *Staphylococcus* was the most abundant, increasing from 37.20% before to 48.69% after probiotic intake, followed by *Cutibacterium*, *Streptococcus*, *Porphyromonas*, *Conchiformibius*, *Acinetobacter*, *Lautropia*, *Arcanobacterium*, *Sphingomonas*, and *Lactobacillus*. At the species level, 886 species were identified, with the top ten species comprising 65.04% of the overall microbiota. *Staphylococcus pseudintermedius* was the most abundant, accounting for 30.29% before and 27.70% after probiotic intake, followed by *Cutibacterium acnes*, *Staphylococcus coagulans*, *Streptococcus canis*, *Staphylococcus capitis*, *Conchiformibius steedae*, *Lautropia mirabilis*, *Arcanobacterium wilhelmae*, *Acinetobacter indicus* CIP 110367, and *Porphyromonas cangingivalis*.

Subsequent non-parametric statistical analysis using the matched-pairs Wilcoxon signed-rank test was conducted to compare relative abundance differences in skin microbiota composition before and after LCP treatment. The results indicated a decreasing trend (*p* < 0.1) in *Enterobacteriaceae* at the family level, *Duncaniella*, *Escherichia*/*Shigella* spp., and *Sphingomonas* at the genera level, and *Shigella flexneri* (*p* < 0.1) at the species level (Figure 5D). For additional reference, Supplementary Tables S9–S12 presented the median (range) of relative abundance for dominant skin bacterial phyla, families, genera, and species, along with *p*-values and FDR-adjusted q-values. Inter-individual variation in skin microbiota composition is similarly visualized in Supplementary Figure S3, which presents relative abundance profiles at multiple taxonomic levels for each dog.

### 3.5. Predicted Functional Pathways of the Skin Microbiota After LCP Treatment

To assess the predicted functional impact of changes in the skin microbiota, KEGG level 2 pathway profiles were inferred using PICRUSt. The analysis focused on immune-related functions and bacterial infections potentially relevant to AD symptoms following LCP treatment. As shown in Figure 5E, a marginal increase (*p* < 0.1) was observed in the predicted abundance of genes associated with the RIG-I-like receptor signaling pathway (ko04622) after LCP treatment. Slight decreases were also observed in predicted gene abundances linked to the Th17 cell differentiation (ko04659), IL-17 signaling (ko04657), and NOD-like receptor signaling (ko04621) pathways. Given that skin damage in AD is often accompanied by secondary infections, we also examined pathways related to bacterial infections. The analysis showed a trend toward reduced predicted functional potential for Shigellosis (ko05131) and *Salmonella* infection (ko05132) pathways following LCP treatment (*p* < 0.1) (Supplementary Table S13).

### 3.6. Correlation Analysis Between Fecal and Skin Microbiota and Atopic Dermatitis Indicators

**Figure 6:**
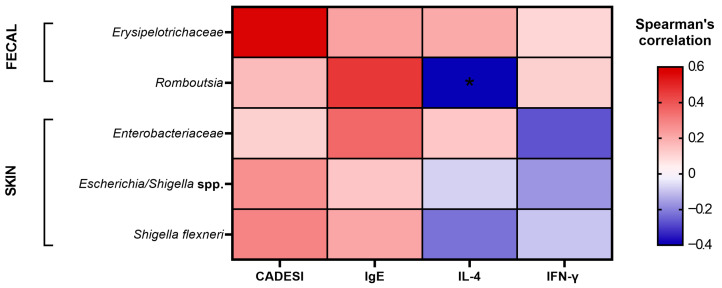
Spearman’s rank correlation analysis between microbial taxa and atopic dermatitis indicators, IgE, and cytokines. Color intensity and direction reflect the strength and direction of the correlation (Spearman’s rho), ranging from −0.4 (blue, negative) to + 0.6 (red, positive). CADESI represents the Canine Atopic Dermatitis Extent and Severity Index. * *p* < 0.05.

Given the complexity and diversity of the gut and skin environments, microbial communities may interact through host-derived metabolites or biochemical substances. To better understand the relationship between these microbial communities and AD indicators, we assessed correlations between previously identified risk-associated taxa and clinical/immunological parameters using Spearman’s rank correlation coefficient. As shown in Figure 6 and Supplementary Table S14, Exploratory correlation analysis suggested that *Erysipelotrichaceae*, *Romboutsia*, *Enterobacteriaceae*, *Escherichia*/*Shigella* spp., and *Shigella flexneri* tended to show positive associations with CADESI scores and serum total IgE levels. In addition, IFN-γ levels appeared to be positively associated with *Erysipelotrichaceae* and *Romboutsia*, while IL-4 secretion showed positive associations with both *Erysipelotrichaceae* and *Enterobacteriaceae*. However, among these, only the negative correlation between *Romboutsia* and IL-4 secretion reached statistical significance (rho = −0.40, *p* = 0.04). These findings highlight potential microbial–immune interactions that warrant further investigation. Supplementary Table S14 provides the detailed correlation coefficients, 95% confidence intervals, and *p*-values to contextualize the strength and statistical certainty of these associations.

### 3.7. Microbial and Metabolic Biomarker Changes in High and Moderate Responders to LCP Treatment

**Figure 7:**
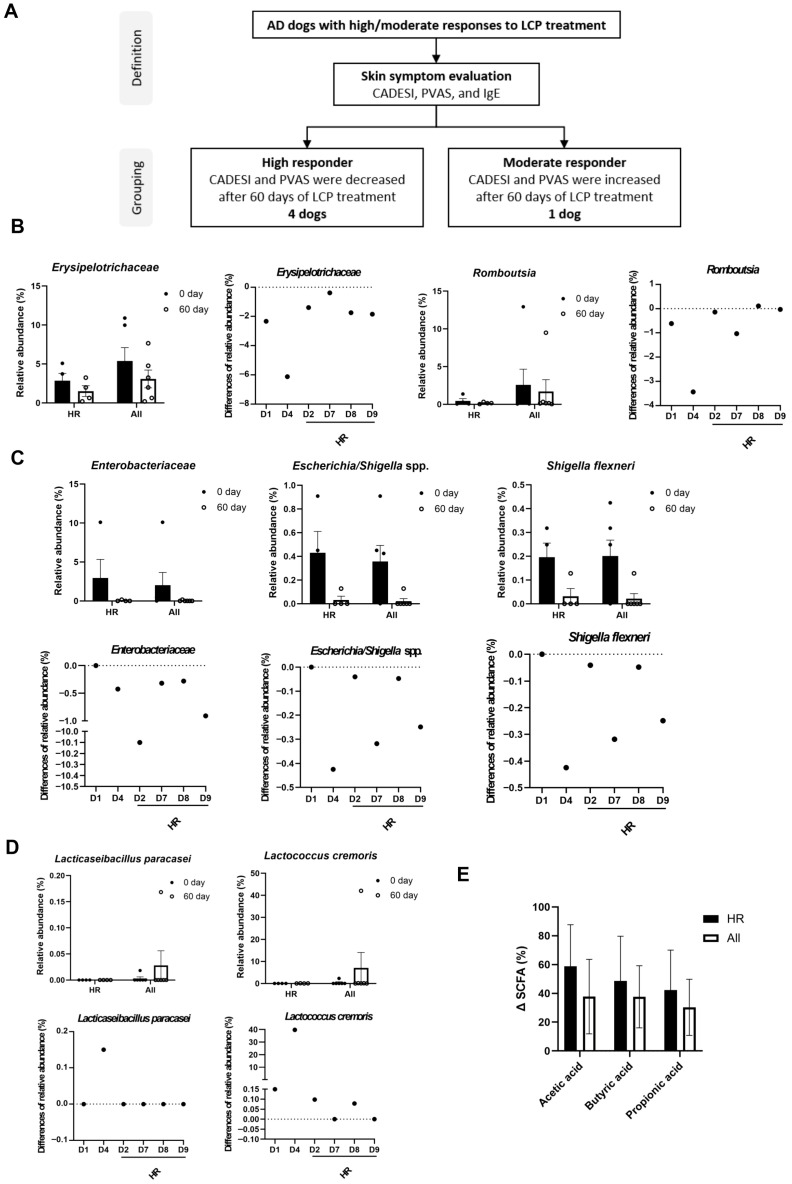
Microbial biomarkers and SCFA levels in high responders (HR) and all treated dogs (All) after 60 days of LCP treatment. (**A**) Criteria for classifying dogs into high responders (HR) and moderate responders (MR). (**B**) Relative abundance and changes in abundance (%) of microbial biomarkers in the feces. (**C**) Relative abundance and changes in abundance (%) of microbial biomarkers in the skin. (**D**) Detection and change in abundance (%) of LCP species in the feces. (**E**) Changes in short-chain fatty acid (SCFA) levels. Data were presented as mean ± SEM (*n* = 4 for HR; *n* = 6 for All). Each dot represented an individual dog. Differences in relative abundance (%) = relative abundance on Day 60 − relative abundance on Day 0.

In our final analysis, we investigated factors influencing the varying responses of individual dogs to LCP treatment for AD and the potential role of the gut and skin microbiome in determining LCP efficacy. Dogs were categorized based on clinical indicators, specifically IgE levels, CADESI scores, and PVAS scores. Since all dogs exhibited a reduction in IgE concentration following LCP treatment, CADESI and PVAS scores were primarily used for classification. Dogs that exhibited reductions in both CADESI and PVAS scores after LCP treatment were classified as high responders (HR), resulting in four dogs meeting this criterion. In contrast, dogs that showed increased scores in both CADESI and PVAS were categorized as moderate responders (MR). One dog exhibited mixed trends and was not grouped in either category. Subsequent comparisons were made between these high-responder dogs (*n* = 4) and the overall cohort (*n* = 6). (Figure 7A).

As shown in Figure 7B, after 60 days of LCP treatment, the relative abundance of *Erysipelotrichaceae* in the fecal microbiota decreased in both groups. Notably, the reduction in *Erysipelotrichaceae* was more pronounced in the HR group compared to the overall population. Conversely, the genus *Romboutsia* exhibited a slight increase in the HR group, whereas a decrease was observed in the overall cohort. In the skin microbiota analysis (Figure 7C), the change in relative abundance indicated that the reductions in *Enterobacteriaceae*, *Escherichia*/*Shigella* spp., and *Shigella flexneri* were greater in the HR group compared to the overall cohort.

Further analysis of the two identified probiotic strains, *Lc. cremoris* subsp. *cremoris* and *L. paracasei* subsp. *paracasei*, in fecal samples revealed that their relative abundance was higher in the overall group compared to the HR group (Figure 7D). Additionally, SCFA levels were analyzed, showing an increasing trend in acetic acid, butyric acid, and propionic acid in the HR group compared to the overall population. Taken together, these findings suggested that not only the relative abundance of LCP but also SCFA levels may contribute to the regulation of AD symptoms (Figure 7E).

## 4. Discussion

This study systematically explores the therapeutic efficacy of a specific probiotic combination (LCP) for managing CAD, a common and persistent allergic skin condition in dogs. The findings provide strong evidence of significant clinical improvement following a 60-day LCP treatment, highlighting its potential as a novel therapeutic approach. Notably, 75% of the canine participants showed a marked reduction in symptom severity. Chronic pruritus in dogs compromises skin health and induces psychological distress, often leading to abnormal behaviors [49]. Thus, alleviating pruritic behavior not only reduces the risk of skin damage and secondary infections but also significantly enhances the overall quality of life in affected dogs [50].

Quantitative analysis of serum cytokines and total IgE levels revealed a progressive decrease in IgE concentrations with prolonged probiotic intake, reaching statistical significance by day 60. Additionally, a decrease in IL-4 levels and an increase in IFN-γ levels were observed in 5 out of 8 treated dogs, corroborating our previous findings from an allergic dermatitis mouse model [27]. Elevated IL-4 levels contribute to increased IgE synthesis, triggering allergic sensitization and exacerbating AD symptoms, whereas reduced IFN-γ levels impair regulatory mechanisms that normally suppress Th2 activity and IgE production [51]. This dysregulation perpetuates inflammation, further aggravating AD symptoms [52,53]. The observed reduction in IgE levels and modulation of the Th1/Th2 balance suggest that LCP may have potential in supporting the management of skin symptoms and pruritic behavior.

The interplay between gut and skin microbiota in patients with AD has garnered significant interest in recent research. In this study, β-diversity was assessed using Bray–Curtis dissimilarity and visualized through Principal Coordinates Analysis (PCoA). Along with α-diversity measures, the results indicated that LCP treatment did not significantly alter the core fecal or skin microbiota composition in treated dogs. However, compositional changes were observed in the fecal microbiota, including a decrease in the relative abundance of specific taxa, leading to reduced evenness and overall diversity. Reduced fecal microbial diversity has been associated with dysbiosis in various allergic and inflammatory diseases, including atopic dermatitis, in both humans [54] and dogs [20,55,56]. A less diverse microbiota may compromise gut barrier integrity and impair immune regulation, increasing the risk of translocation of microbial products or toxins into systemic circulation. These gut-derived signals can contribute to systemic inflammation and promote Th2-skewed immune responses, which are characteristic of AD and may exacerbate skin barrier dysfunction and inflammatory lesions [57,58].

Current literature presents conflicting conclusions regarding the impact of AD on gut and skin microbiota diversity. While numerous studies report that patients with AD exhibit reduced gut microbiota diversity compared to healthy controls [59,60,61], other findings suggest no significant differences between the two groups [62,63]. Skin microbiota diversity is influenced by various external factors, including environmental conditions, humidity, skin structure, and sebum distribution [64]. For instance, sebum secretion may promote the growth of lipophilic bacteria, potentially increasing microbiota diversity in drier skin environments [65], whereas a compromised skin barriers could facilitate microbial colonization due to enhanced environmental exposure or moisture from tissue fluid secretion [22].

Further analysis of dominant bacterial taxa in both the fecal and skin microbiota before and after LCP treatment revealed significant compositional changes. In the fecal microbiota, *Erysipelotrichaceae* showed a notable reduction, while the genus *Romboutsia* exhibited a decreasing trend following LCP treatment. *Erysipelotrichaceae* has been linked to increased *NOD2* gene expression in human models [66]. *NOD2*, a cytosolic pattern-recognition receptor predominantly found in epithelial cells, plays a critical role in pro-inflammatory signaling and Th1 response modulating via toll-like receptors (TLRs) [67,68,69,70]. Previous mouse model studies have demonstrated that *NOD2* gene deletion leads to reduced production of Th2-associated cytokines [71], aligning with our findings that associate *Erysipelotrichaceae* abundance with IL-4 and IgE levels. For *Romboutsia*, prior human studies have reported higher prevalence in atopic dermatitis compared to other allergic conditions [72]. *Romboutsia* has been positively associated with pro-inflammatory cytokines, suggesting its role in influencing Th17 cell differentiation and affecting skin barrier integrity [73,74].

In the skin microbiota, reductions were observed in *Duncaniella*, *Escherichia*/*Shigella* spp., and *Sphingomonas* at the genus level, with a species-level decrease in *S. flexneri* after LCP treatment. *Duncaniella*, formerly classified as S24-7, belongs to the *Muribaculaceae* family and is a key bacterial group in the gut microbiota of laboratory mice [75]. Certain *Duncaniella* species have demonstrated protective effects against DSS-induced intestinal damage [76]. *Sphingomonas*, part of the *Sphingomonadaceae* family, has been found in higher proportions on the skin of healthy cattle in regions with greater rainfall [77,78]. However, the effects of both microbial taxa on canine skin require further investigation.

Regarding *S. flexneri*, this species belongs to the *Shigella* genus within the *Enterobacteriaceae* family. While its role in canine atopic dermatitis remains unclear, human studies suggest that *S. flexneri* activates epithelial cell NLRs, thereby triggering inflammatory responses, particularly via NOD1 recognition. This interaction may enhance NOD1 signaling through NLR family pyrin domain containing 10 (NLRP10) expression in epidermal and dermal fibroblast-like cells, potentially influencing innate immune responses and exhibiting the associations with atopic dermatitis and allergic contact dermatitis [79,80,81,82].

Changes in gut and skin microbial composition were accompanied by notable shifts in predicted microbial functions based on KEGG pathway analysis following LCP treatment. Functional prediction suggested a trend toward higher relative abundance of KEGG genes involved in short-chain fatty acid (SCFA) biosynthesis pathways in the gut, which is consistent with our direct measurements. Higueras et al. (2021) reported mean fecal concentrations of 3852, 3049, and 1634 µmol/g dry matter for acetate, propionate, and butyrate, respectively, in healthy dogs [83]. In comparison, this study observed lower levels of these SCFAs in dogs with atopic dermatitis, potentially reflecting an underlying microbial or metabolic imbalance. The observed increasing trend in SCFA concentrations following LCP treatment may reflect a shift toward a healthier gut metabolic profile. Notably, most dogs exhibited elevated levels of acetate, propionate, and butyrate after 60 days of LCP administration. These increases may result from the metabolic activity of the probiotic strains themselves, which can ferment carbohydrates into SCFAs, and from indirect effects on indigenous gut microbes. LCP may help reshape the gut microbiota to favor the proliferation of SCFA-producing taxa, thereby enhancing SCFA biosynthesis. These metabolites, in turn, are known to support gut epithelial integrity, regulate immune responses, and contribute to skin homeostasis via the gut–skin axis. Previous research has demonstrated that gut-derived SCFAs influence epidermal keratinocytes by modifying their metabolism, enhancing skin barrier function and reducing allergen penetration [84]. Furthermore, SCFAs act as histone deacetylase (HDAC) inhibitors, decreasing the production of pro-inflammatory cytokines via suppressing the NF-κB pathway. This HDAC inhibition also contributes to regulatory T (Treg) cell regulation and keratinocyte differentiation, further promoting to skin health [85,86,87].

Regarding immune-related pathways, PICRUSt-based prediction suggested a lower relative abundance of KEGG orthologs related to the NOD-like receptor (NLR) signaling pathway in both fecal and skin microbiota, and a higher predicted abundance of genes associated with the retinoic acid-inducible gene (RIG)-I-like receptor (RLR) signaling pathway in the skin after LCP treatment. *Erysipelotrichaceae* and *S. flexneri*, which were reduced in the fecal and skin microbiota, respectively, following LCP treatment, are known activators of NOD-like receptors (NLRs), potentially amplifying inflammatory responses in skin lesions and exacerbating symptoms of atopic dermatitis [88,89]. Conversely, RLR signaling may play a role in regulating the Th1/Th2 balance and modulating inflammatory responses [90]. These microbial shifts may contribute to the observed reductions in serum IgE and IL-4 levels. Additionally, predicted functional changes in microbial pathways related to *Salmonella* infection and shigellosis were noted. These findings highlight a potential link between microbiota composition and immune function, suggesting that LCP may modulate atopic dermatitis via microbiome-mediated immunoregulatory mechanisms.

The responses to probiotic treatment among dogs with AD exhibited considerable heterogeneity. In our analysis, dogs with high responsiveness to LCP demonstrated a greater reduction in the relative abundance of *Erysipelotrichaceae*, *Romboutsia*, *Enterobacteriaceae*, *Escherichia*/*Shigella* spp., and *S. flexneri* following LCP treatment. This microbial shift correlated with improvement in CADESI scores, IgE levels, and Th1/Th2 balance in serum, likely due to the observed associations between specific bacterial taxa and AD indicators. These findings reflect correlations rather than causal relationships, and further studies with controlled interventions will be necessary to confirm these associations and elucidate underlying mechanisms. Furthermore, higher SCFA levels in high-responder dogs suggest that the beneficial effects of SCFA including strengthening the skin barrier, reducing inflammation, and regulating immune responses, may contribute to symptom relief in AD. In contrast, dogs with low responsiveness to LCP exhibited a less pronounced decrease in *Erysipelotrichaceae*, *Enterobacteriaceae*, *Escherichia*/*Shigella* spp., and *S. flexneri*, as well as lower SCFA levels after LCP treatment. Further investigation is needed to clarify the potential roles of these microorganisms in AD pathogenesis and the interaction of LCP and SCFA production in the gut of dogs with AD [84,85,86,87]. Clinical studies on probiotics indicate that individual factors such as diet, age, physiological condition, immune response, and the composition of indigenous gut microbiota can significantly influence probiotic efficacy, leading to variable treatment outcomes [91]. Given these complexities, further research is warranted to identify the specific factors affecting LCP efficacy in managing canine AD. Recognizing these variability determinants could help optimize probiotic interventions to enhance therapeutic outcomes in canine AD.

There were several limitations in this study. First, it was conducted as an open-label, single-arm pilot trial without a control group and included a small sample size, which restricts the statistical power and limits the generalizability of the findings. The absence of a placebo or control group makes it difficult to exclude the possibility that observed improvements were due to natural disease fluctuations, seasonal variation, measurement variability, or subjective bias in CADESI and PVAS scoring. While single-arm designs are commonly used in early-phase exploratory studies, particularly when ethical or logistical challenges make randomization difficult [92]. Accordingly, all findings have been interpreted within an exploratory framework, and future randomized controlled trials will be necessary to validate these preliminary results and assess the efficacy of LCP more definitively. Additionally, five out of eight dogs had CADESI scores below 30, indicating mild disease severity at baseline; except for using Favrot’s criteria for CAD diagnosis as recruitment criteria, future studies should therefore consider setting a minimum CADESI threshold (e.g., ≥35, moderate to severe) to better evaluate therapeutic efficacy. Second, due to the small sample size and exploratory nature of this study, non-parametric statistical methods were applied to evaluate changes in microbial relative abundances. Future studies with larger cohorts should incorporate compositional data analysis approaches, such as centered log-ratio (CLR) transformation, or advanced statistical frameworks like ANCOM-BC2 and MaAsLin3, which are specifically designed to account for compositionality and provide more robust differential abundance testing. Third, although our study demonstrated that the administered species *Lactococcus cremoris* was more frequently detected in fecal samples at the end of the intervention, this observation reflects its presence during the LCP administration period rather than confirmed colonization. The absence of post-treatment follow-up sampling limits our ability to assess the persistence or integration of the strain within the gut microbiota. Future studies incorporating extended washout periods and post-intervention sampling will be necessary to assess the persistence and true colonization dynamics of the administered probiotic strains. Forth, interpretation of microbial taxa with low relative abundance or inconsistent detection across subjects should be made with caution. Several taxa identified as potentially relevant were present only in a subset of dogs, raising the possibility that these findings reflect inter-individual variability or stochastic detection rather than true biological significance. Additionally, low-abundance features are more susceptible to sequencing depth variation and compositional bias, which can amplify noise in diversity and differential abundance estimates. Therefore, results involving rare taxa should be interpreted cautiously and validated in future studies using larger sample sizes and deeper sequencing methods. Lastly, this study utilized PICRUSt to infer microbial functional potential based on 16S rRNA gene sequences. However, this predictive approach does not measure actual gene expression, protein activity, or host–microbe interactions. Therefore, the observed changes in microbial functions, particularly those related to metabolism or immune signaling, should be interpreted as computational predictions rather than direct evidence of biological activity. Future studies incorporating metagenomic, transcriptomic, or host immunological analyses will be essential to validate and further investigate these functional hypotheses.

## 5. Conclusions

In summary, supplementation with the LCP probiotic combination improved clinical symptoms, reduced pruritus, and modulated immune indicators in dogs with atopic dermatitis, suggesting potential therapeutic benefits. These improvements were accompanied by compositional shifts in both fecal and skin microbiota, characterized by decreased abundances of *Erysipelotrichaceae*, *Romboutsia*, *Enterobacteriaceae*, *Escherichia*/*Shigella* spp., and *S. flexneri*, along with an increase in *Lactococcus cremoris*. Functional prediction indicated that LCP may enhance SCFA synthesis and modulate immune-related pathways such as NLR signaling, leading to elevated SCFA levels and reduced serum IgE and IL-4 concentrations. Additionally, modulation through the gut–skin axis may influence systemic immune responses, contributing to the stabilization of skin microbiota composition and a reduced capacity for adherence and invasion by environmental microbes and pathogens. Collectively, these findings support that LCP alleviates atopic symptoms through gut–skin axis–mediated microbial and immune modulation and highlight its potential as an adjunctive microbiome-based strategy for managing canine atopic dermatitis.

## Data Availability

The authors confirm that the data supporting the findings of this study are available within the article and its Supplementary Materials. Raw data that support the findings of this study are available from the corresponding author upon reasonable request.

## Supplementary Materials

The following supporting information can be downloaded at https://www.mdpi.com/article/10.3390/ani15213098/s1, Figure S1. Clinical assessment of atopic dermatitis severity over time following LCP treatment; Figure S2. Stacked bar plots showing the relative abundances of the dominant fecal bacterial taxa in individual dogs with atopic dermatitis at (A) phylum, (B) family, (C) genus, and (D) species levels; Figure S3. Stacked bar plots showing the relative abundances of the dominant skin bacterial taxa in individual dogs with atopic dermatitis at (A) phylum, (B) family, (C) genus, and (D) species levels; Table S1. Demographic characteristics of recruitment dogs; Table S2. CADESI scores and PVAS scores of recruitment dogs before LCP treatment; Table S3. Estimated marginal means, confidence intervals, and overall time effects for CADESI and PVAS scores in dogs with atopic dermatitis (AD) before, during, and after LCP treatment; Table S4. Relative abundance of dominant fecal bacterial phyla before and after 60 days of LCP treatment in dogs with atopic dermatitis; Table S5. Relative abundance of dominant fecal bacterial families before and after 60 days of LCP treatment in dogs with atopic dermatitis; Table S6. Relative abundance of dominant fecal bacterial genera before and after 60 days of LCP treatment in dogs with atopic dermatitis; Table S7. Relative abundance of dominant fecal bacterial species before and after 60 days of LCP treatment in dogs with atopic dermatitis; Table S8. Relative abundance of carbohydrate metabolism and immune system associated pathways in the fecal microbiome of dogs with atopic dermatitis before and after 60 days of LCP treatment; Table S9. Relative abundance of dominant skin bacterial phyla before and after 60 days of LCP treatment in dogs with atopic dermatitis; Table S10. Relative abundance of dominant skin bacterial families before and after 60 days of LCP treatment in dogs with atopic dermatitis; Table S11. Relative abundance of dominant skin bacterial genera before and after 60 days of LCP treatment in dogs with atopic dermatitis; Table S12. Relative abundance of dominant skin bacterial species before and after 60 days of LCP treatment in dogs with atopic dermatitis; Table S13. Relative abundance of immune system and bacterial infectious diseases associated pathways in the skin microbiome of dogs with atopic dermatitis before and after 60 days of LCP treatment; Table S14. Spearman correlation coefficients (r), 95% confidence intervals (CI), and *p*-values between selected bacterial taxa and immunological indicators (IgE, IL-4, and IFN-γ).

## Abbreviations

The following abbreviations are used in this manuscript:

ASVs, amplicon sequence variants; CAD, canine atopic dermatitis; CADESI, Canine Atopic Dermatitis Extent and Severity Index; CBC, complete blood count; DPBS, Dulbecco’s phosphate-buffered saline; ELISA, enzyme-linked immunosorbent assay; FBS, fetal bovine serum; HPLC, high-performance liquid chromatography; HR, high responders; HDACs, histone deacetylases; IACUC, Institutional Animal Care and Use Committee; ICADA, International Consortium on Allergic Diseases in Animals; IFN-γ, interferon-γ; IgE, immunoglobulin E; IL, interleukin; KEGG, Kyoto Encyclopedia of Genes and Genomes; MR, moderate responders; NOD, nucleotide-binding oligomerization domain; NLR, NOD-like receptor; NLRP10, NLR family pyrin domain containing 10; PBMC, peripheral blood mononuclear cells; PCoA, principal coordinates analysis; PICRUSt, Phylogenetic Investigation of Communities by Reconstruction of Unob-served States; PVAS, Pruritus Visual Analogue Scale; RIG, retinoic acid-inducible gene; RLR, RIG-I-like receptor; RPMI, Roswell Park Memorial Institute; TLRs, toll-like receptors; Treg, regulatory T cells; SCFA, short-chain fatty acid; Th1 cells, type 1 T helper cells; Th2 cells, type 2 T helper cells.

## References

[1] Widorn L., Zabolotski Y., Mueller R.S. (2024). A prospective study evaluating the correlation between local weather conditions, pollen counts and pruritus of dogs with atopic dermatitis. Vet. Dermatol..

[2] Gedon N.K.Y., Mueller R.S. (2018). Atopic dermatitis in cats and dogs: A difficult disease for animals and owners. Clin. Transl. Allergy.

[3] Outerbridge C.A., Jordan T.J. (2021). Current knowledge on canine atopic dermatitis: Pathogenesis and treatment. Adv. Small Anim. Care.

[4] Couceiro G.A., Ribeiro S.M.M., Monteiro M.M., Meneses A.M.C., Sousa S.K.S., Coutinho L.N. (2021). Prevalence of canine atopic dermatitis at the Veterinary Hospital of the “Universidade Federal Rural da Amazônia” in Belém/Pará, Brazil. Pesqui. Vet. Bras..

[5] Humeniuk P., Dubiela P., Hoffmann-Sommergruber K. (2017). Dendritic cells and their role in allergy: Uptake, proteolytic processing and presentation of allergens. Int. J. Mol. Sci..

[6] Marsella R., Sousa C.A., Gonzales A.J., Fadok V.A. (2012). Current understanding of the pathophysiologic mechanisms of canine atopic dermatitis. J. Am. Vet. Med. Assoc..

[7] Nguyen H.L., Anderson K.R., Tollefson M.M. (2019). New and emerging therapies for pediatric atopic dermatitis. Paediatr. Drugs.

[8] Cabanillas B., Novak N. (2016). Atopic dermatitis and filaggrin. Curr. Opin. Immunol..

[9] McAleer M.A., Irvine A.D. (2013). The multifunctional role of filaggrin in allergic skin disease. J. Allergy Clin. Immunol..

[10] Li S., Villarreal M., Stewart S., Choi J., Ganguli-Indra G., Babineau D.C., Philpot C., David G., Yoshida T., Boguniewicz M. (2017). Altered composition of epidermal lipids correlates with *Staphylococcus aureus* colonization status in atopic dermatitis. Br. J. Dermatol..

[11] Shimada K., Yoon J.S., Yoshihara T., Iwasaki T., Nishifuji K. (2009). Increased transepidermal water loss and decreased ceramide content in lesional and non-lesional skin of dogs with atopic dermatitis. Vet. Dermatol..

[12] Gonzales A.J., Bowman J.W., Fici G.J., Zhang M., Mann D.W., Mitton-Fry M. (2014). Oclacitinib (APOQUEL^®^) is a novel Janus kinase inhibitor with activity against cytokines involved in allergy. J. Vet. Pharmacol. Ther..

[13] Bağci I.S., Ruzicka T. (2018). IL-31: A new key player in dermatology and beyond. J. Allergy Clin. Immunol..

[14] Chrobak-Chmiel D., Golke A., Kwiecień E., Biegańska M.J., Dembele K., Dziekiewicz-Mrugasiewicz M., Czopowicz M., Kizerwetter-Świda M., Rzewuska M. (2023). Is vitamin D3 a worthy supplement protecting against secondary infections in dogs with atopic dermatitis?. Pathogens.

[15] De Pessemier B., Grine L., Debaere M., Maes A., Paetzold B., Callewaert C. (2021). Gut-skin axis: Current knowledge of the interrelationship between microbial dysbiosis and skin conditions. Microorganisms.

[16] Pfefferle P.I., Keber C.U., Cohen R.M., Garn H. (2021). The hygiene hypothesis-learning from but not living in the past. Front. Immunol..

[17] Dou J., Zeng J., Wu K., Tan W., Gao L., Lu J. (2019). Microbiosis in pathogenesis and intervention of atopic dermatitis. Int. Immunopharmacol..

[18] Fujimura K.E., Sitarik A.R., Havstad S., Lin D.L., Levan S., Fadrosh D., Panzer A.R., LaMere B., Rackaityte E., Lukacs N.W. (2016). Neonatal gut microbiota associates with childhood multisensitized atopy and T cell differentiation. Nat. Med..

[19] Fang Z., Li L., Zhang H., Zhao J., Lu W., Chen W. (2021). Gut microbiota, probiotics, and their interactions in prevention and treatment of atopic dermatitis: A review. Front. Immunol..

[20] Rostaher A., Morsy Y., Favrot C., Unterer S., Schnyder M., Scharl M., Fischer N.M. (2022). Comparison of the gut microbiome between atopic and healthy dogs-preliminary data. Animals.

[21] Sinkko H., Lehtimäki J., Lohi H., Ruokolainen L., Hielm-Björkman A. (2023). Distinct healthy and atopic canine gut microbiota is influenced by diet and antibiotics. R. Soc. Open Sci..

[22] Ellis S.R., Nguyen M., Vaughn A.R., Notay M., Burney W.A., Sandhu S., Sivamani R.K. (2019). The skin and gut microbiome and its role in common dermatologic conditions. Microorganisms.

[23] Kwon H.K., Lee C.G., So J.S., Chae C.S., Hwang J.S., Sahoo A., Nam J.H., Rhee J.H., Hwang K.C., Im S.H. (2010). Generation of regulatory dendritic cells and CD_4_+Foxp_3_+ T cells by probiotics administration suppresses immune disorders. Proc. Natl. Acad. Sci. USA.

[24] Shin J.H., Chung M.J., Seo J.G. (2016). A multistrain probiotic formulation attenuates skin symptoms of atopic dermatitis in a mouse model through the generation of CD_4_^+^ Foxp_3_^+^ T cells. Food Nutr. Res..

[25] Kim W.K., Jang Y.J., Han D.H., Seo B., Park S., Lee C.H., Ko G. (2019). Administration of *Lactobacillus fermentum* KBL375 causes taxonomic and functional changes in gut microbiota leading to improvement of atopic dermatitis. Front. Mol. Biosci..

[26] Cauquil M., Olivry T. (2025). Immunomodulating effects of heat-killed *Lactobacillus rhamnosus* and *Lactobacillus reuteri* on peripheral blood mononuclear cells from healthy dogs. Vet. Sci..

[27] Chen H.Y., Chen Y.T., Li K.Y., Huang H.W., Lin Y.C., Chen M.J. (2022). A heat-killed probiotic mixture regulates immune T cells balance and IgE production in house dust mite extraction-induced atopic dermatitis mice. Microorganisms.

[28] Favrot C., Steffan J., Seewald W., Picco F. (2010). A prospective study on the clinical features of chronic canine atopic dermatitis and its diagnosis. Vet. Dermatol..

[29] Terada Y., Nagata M., Murayama N., Nanko H., Furue M. (2011). Clinical comparison of human and canine atopic dermatitis using human diagnostic criteria (Japanese Dermatological Association, 2009): Proposal of provisional diagnostic criteria for canine atopic dermatitis. J. Dermatol..

[30] Olivry T., Saridomichelakis M., Nuttall T., Bensignor E., Griffin C.E., Hill P.B., International Committee on Allergic Diseases of Animals (ICADA) (2014). Validation of the canine atopic dermatitis extent and severity index (CADESI)-4, a simplified severity scale for assessing skin lesions of atopic dermatitis in dogs. Vet. Dermatol..

[31] Rybnícek J., Lau-Gillard P.J., Harvey R., Hill P.B. (2009). Further validation of a pruritus severity scale for use in dogs. Vet. Dermatol..

[32] Matsuki T., Watanabe K., Fujimoto J., Kado Y., Takada T., Matsumoto K., Tanaka R. (2004). Quantitative PCR with 16S rRNA-gene-targeted species-specific primers for analysis of human intestinal bifidobacteria. Appl. Environ. Microbiol..

[33] Callahan B.J., McMurdie P.J., Rosen M.J., Han A.W., Johnson A.J., Holmes S.P. (2016). DADA2: High-resolution sample inference from Illumina amplicon data. Nat. Methods..

[34] Quin C., Estaki M., Vollman D.M., Barnett J.A., Gill S.K., Gibson D.L. (2018). Probiotic supplementation and associated infant gut microbiome and health: A cautionary retrospective clinical comparison. Sci. Rep..

[35] Camacho C., Coulouris G., Avagyan V., Ma N., Papadopoulos J., Bealer K., Madden T.L. (2009). BLAST+: Architecture and applications. BMC Bioinform..

[36] Bolyen E., Rideout J.R., Dillon M.R., Bokulich N.A., Abnet C.C., Al-Ghalith G.A., Alexander H., Alm E.J., Arumugam M., Asnicar F. (2019). Reproducible, interactive, scalable and extensible microbiome data science using QIIME 2. Nat. Biotechnol..

[37] Katoh K., Standley D.M. (2013). MAFFT multiple sequence alignment software version 7: Improvements in performance and usability. Mol. Biol. Evol..

[38] Balvočiūtė M., Huson D.H. (2017). SILVA, RDP, Greengenes, NCBI and OTT—How do these taxonomies compare?. BMC Genom..

[39] Gyarmati P., Kjellander C., Aust C., Song Y., Öhrmalm L., Giske C.G. (2016). Metagenomic analysis of bloodstream infections in patients with acute leukemia and therapy-induced neutropenia. Sci. Rep..

[40] Hong X., Chen J., Liu L., Wu H., Tan H., Xie G., Xu Q., Zou H., Yu W., Wang L. (2016). Metagenomic sequencing reveals the relationship between microbiota composition and quality of Chinese rice wine. Sci. Rep..

[41] Triplett J., Ellis D., Braddock A., Roberts E., Ingram K., Perez E., Short A., Brown D., Hutzley V., Webb C. (2020). Temporal and region-specific effects of sleep fragmentation on gut microbiota and intestinal morphology in Sprague Dawley rats. Gut Microbes.

[42] Shin J., Noh J.R., Choe D., Lee N., Song Y., Cho S., Kang E.J., Go M.J., Ha S.K., Chang D.H. (2021). Ageing and rejuvenation models reveal changes in key microbial communities associated with healthy ageing. Microbiome.

[43] Roelands J., Kuppen P.J.K., Ahmed E.I., Mall R., Masoodi T., Singh P., Monaco G., Raynaud C., de Miranda N.F., Ferraro L. (2023). An integrated tumor, immune and microbiome atlas of colon cancer. Nat. Med..

[44] Bray J.R., Curtis J.T. (1957). An ordination of the upland forest communities of southern Wisconsin. Ecol. Monogr..

[45] Grabrucker S., Marizzoni M., Silajdžić E., Lopizzo N., Mombelli E., Nicolas S., Dohm-Hansen S., Scassellati C., Moretti D.V., Rosa M. (2023). Microbiota from Alzheimer’s patients induce deficits in cognition and hippocampal neurogenesis. Brain.

[46] Wemheuer F., Taylor J.A., Daniel R., Johnston E., Meinicke P., Thomas T., Wemheuer B. (2020). Tax4Fun2: Prediction of habitat-specific functional profiles and functional redundancy based on 16S rRNA gene sequences. Environ. Microbiomes.

[47] Torii T., Kanemitsu K., Wada T., Itoh S., Kinugawa K., Hagiwara A. (2010). Measurement of short-chain fatty acids in human faeces using high-performance liquid chromatography: Specimen stability. Ann. Clin. Biochem..

[48] Creevy K.E., Grady J., Little S.E., Moore G.E., Strickler B.G., Thompson S., Webb J.A. (2019). 2019 AAHA canine life stage guidelines. J. Am. Anim. Hosp. Assoc..

[49] Harvey N.D., Craigon P.J., Shaw S.C., Blott S.C., England G.C. (2019). Behavioural differences in dogs with atopic dermatitis suggest stress could be a significant problem associated with chronic pruritus. Animals.

[50] Linek M., Favrot C. (2010). Impact of canine atopic dermatitis on the health-related quality of life of affected dogs and quality of life of their owners. Vet. Dermatol..

[51] Udoye C.C., Rau C.N., Freye S.M., Almeida L.N., Vera-Cruz S., Othmer K., Korkmaz R.Ü., Clauder A., Lindemann T., Manz R.A. (2022). B-cell receptor physical properties affect relative IgG1 and IgE responses in mouse egg allergy. Mucosal Immunol..

[52] Carballo I., Alonso-Sampedro M., Gonzalez-Conde E., Sanchez-Castro J., Vidal C., Gude F., Gonzalez-Quintela A. (2021). Factors influencing total serum IgE in adults: The role of obesity and related metabolic disorders. Int. Arch. Allergy Immunol..

[53] Chaudhary S.K., Singh S.K., Kumari P., Kanwal S., Soman S.P., Choudhury S., Garg S.K. (2019). Alterations in circulating concentrations of IL-17, IL-31 and total IgE in dogs with atopic dermatitis. Vet. Dermatol..

[54] Lee S.Y., Lee E., Park Y.M., Hong S.J. (2018). Microbiome in the Gut-Skin Axis in Atopic Dermatitis. Allergy Asthma Immunol. Res..

[55] Thomsen M., Künstner A., Wohlers I., Olbrich M., Lenfers T., Osumi T., Shimazaki Y., Nishifuji K., Ibrahim S.M., Watson A. (2023). A comprehensive analysis of gut and skin microbiota in canine atopic dermatitis in Shiba Inu dogs. Microbiome.

[56] Song H., Mun S.H., Han D.W., Kang J.H., An J.U., Hwang C.Y., Cho S. (2025). Probiotics ameliorate atopic dermatitis by modulating the dysbiosis of the gut microbiota in dogs. BMC Microbiol..

[57] Seite S., Bieber T. (2015). Barrier function and microbiotic dysbiosis in atopic dermatitis. Clin. Cosmet. Investig. Dermatol..

[58] Kim J.E., Kim H.S. (2019). Microbiome of the Skin and Gut in Atopic Dermatitis (AD): Understanding the pathophysiology and finding novel management strategies. J. Clin. Med..

[59] Reddel S., Del Chierico F., Quagliariello A., Giancristoforo S., Vernocchi P., Russo A., Fiocchi A., Rossi P., Putignani L., El Hachem M. (2019). Gut microbiota profile in children affected by atopic dermatitis and evaluation of intestinal persistence of a probiotic mixture. Sci. Rep..

[60] Sugita K., Shima A., Takahashi K., Ishihara G., Kawano K., Ohmori K. (2023). Pilot evaluation of a single oral fecal microbiota transplantation for canine atopic dermatitis. Sci. Rep..

[61] Ye S., Yan F., Wang H., Mo X., Liu J., Zhang Y., Li H., Chen D. (2021). Diversity analysis of gut microbiota between healthy controls and those with atopic dermatitis in a Chinese population. J. Dermatol..

[62] Uchiyama J., Osumi T., Mizukami K., Fukuyama T., Shima A., Unno A., Takemura-Uchiyama I., Une Y., Murakami H., Sakaguchi M. (2022). Characterization of the oral and faecal microbiota associated with atopic dermatitis in dogs selected from a purebred Shiba Inu colony. Lett. Appl. Microbiol..

[63] Wang Y., Hou J., Tsui J.C.C., Wang L., Zhou J., Chan U.K., Lo C.J.Y., Siu P.L.K., Loo S.K.F., Tsui S.K.W. (2023). Unique gut microbiome signatures among adult patients with moderate to severe atopic dermatitis in southern Chinese. Int. J. Mol. Sci..

[64] Skowron K., Bauza-Kaszewska J., Kraszewska Z., Wiktorczyk-Kapischke N., Grudlewska-Buda K., Kwiecińska-Piróg J., Wałecka-Zacharska E., Radtke L., Gospodarek-Komkowska E. (2021). Human Skin Microbiome: Impact of Intrinsic and Extrinsic Factors on Skin Microbiota. Microorganisms.

[65] Swaney M.H., Nelsen A., Sandstrom S., Kalan L.R. (2023). Sweat and Sebum Preferences of the Human Skin Microbiota. Microbiol. Spectr..

[66] Turpin W., Bedrani L., Espin-Garcia O., Xu W., Silverberg M.S., Smith M.I., Garay J.A.R., Lee S.H., Guttman D.S., Griffiths A. (2020). Associations of NOD2 polymorphisms with *Erysipelotrichaceae* in stool of in healthy first degree relatives of Crohn’s disease subjects. BMC Med. Genet..

[67] Duan W., Mehta A.K., Magalhaes J.G., Ziegler S.F., Dong C., Philpott D.J., Croft M. (2010). Innate signals from Nod2 block respiratory tolerance and program TH2-driven allergic inflammation. J. Allergy Clin. Immunol..

[68] Girardin S.E., Boneca I.G., Viala J., Chamaillard M., Labigne A., Thomas G., Philpott D.J., Sansonetti P.J. (2003). Nod2 is a general sensor of peptidoglycan through muramyl dipeptide (MDP) detection. J. Biol. Chem..

[69] Liu T., Zhang L., Joo D., Sun S.C. (2017). NF-κB signaling in inflammation. Signal Transduct. Target. Ther..

[70] Maeda S., Hsu L.C., Liu H., Bankston L.A., Iimura M., Kagnoff M.F., Eckmann L., Karin M. (2005). Nod2 mutation in Crohn’s disease potentiates NF-κB activity and IL-1ß processing. Science.

[71] Corridoni D., Rodriguez-Palacios A., Di Stefano G., Di Martino L., Antonopoulos D.A., Chang E.B., Arseneau K.O., Pizarro T.T., Cominelli F. (2017). Genetic deletion of the bacterial sensor NOD2 improves murine Crohn’s disease-like ileitis independent of functional dysbiosis. Mucosal Immunol..

[72] Su Y.J., Luo S.D., Hsu C.Y., Kuo H.C. (2021). Differences in gut microbiota between allergic rhinitis, atopic dermatitis, and skin urticaria: A pilot study. Medicine.

[73] Li R., Yao Y., Gao P., Bu S. (2021). The therapeutic efficacy of curcumin vs. metformin in modulating the gut microbiota in NAFLD rats: A comparative study. Front. Microbiol..

[74] Wang H.G., Zhang M.N., Wen X., He L., Zhang M.H., Zhang J.L., Yang X.Z. (2022). Cepharanthine ameliorates dextran sulphate sodium-induced colitis through modulating gut microbiota. Microb. Biotechnol..

[75] Lagkouvardos I., Lesker T.R., Hitch T.C.A., Gálvez E.J.C., Smit N., Neuhaus K., Wang J., Baines J.F., Abt B., Stecher B. (2019). Sequence and cultivation study of Muribaculaceae reveals novel species, host preference, and functional potential of this yet undescribed family. Microbiome.

[76] Chang C.S., Liao Y.C., Huang C.T., Lin C.M., Cheung C.H.Y., Ruan J.W., Yu W.H., Tsai Y.T., Lin I.J., Huang C.H. (2021). Identification of a gut microbiota member that ameliorates DSS-induced colitis in intestinal barrier enhanced Dusp6-deficient mice. Cell Rep..

[77] Glaeser S.P., Kämpfer P., Rosenberg E., DeLong E.F., Lory S., Stackebrandt E., Thompson F. (2014). The family *Sphingomonadaceae*. The Prokaryotes: Alphaproteobacteria and Betaproteobacteria.

[78] Gamage H.K., Vuong D., Minns S.A., Chen R., Piggott A.M., Lacey E., Paulsen I.T. (2022). The composition and natural variation of the skin microbiota in healthy Australian cattle. Res. Sq..

[79] Imamura R., Wang Y., Kinoshita T., Suzuki M., Noda T., Sagara J., Taniguchi S., Okamoto H., Suda T. (2010). Anti-inflammatory activity of PYNOD and its mechanism in humans and mice. J. Immunol..

[80] Lautz K., Damm A., Menning M., Wenger J., Adam A.C., Zigrino P., Kremmer E., Kufer T.A. (2012). NLRP 10 enhances Shigella-induced pro-inflammatory responses. Cell. Microbiol..

[81] Mirza N., Sowa A.S., Lautz K., Kufer T.A. (2019). NLRP10 affects the stability of abin-1 to control inflammatory responses. J. Immunol..

[82] Fritz J.H., Le Bourhis L., Sellge G., Magalhaes J.G., Fsihi H., Kufer T.A., Collins C., Viala J., Ferrero R.L., Girardin S.E. (2007). Nod1-mediated innate immune recognition of peptidoglycan contributes to the onset of adaptive immunity. Immunity.

[83] Higueras C., Rey A.I., Escudero R., Díaz-Regañón D., Rodríguez-Franco F., García-Sancho M., Agulla B., Sainz A. (2021). Short-chain and total fatty acid profile of faeces or plasma as predictors of food-responsive enteropathy in dogs: A preliminary study. Animals.

[84] Trompette A., Pernot J., Perdijk O., Alqahtani R.A.A., Santo Domingo J., Camacho-Muñoz D., Wong N.C., Kendall A.C., Wiederkehr A., Nicod L.P. (2022). Gut-derived short-chain fatty acids modulate skin barrier integrity by promoting keratinocyte metabolism and differentiation. Mucosal Immunol..

[85] Aoyama M., Kotani J., Usami M. (2010). Butyrate and propionate induced activated or non-activated neutrophil apoptosis via HDAC inhibitor activity but without activating GPR-41/GPR-43 pathways. Nutrition.

[86] Carrion S.L., Sutter C.H., Sutter T.R. (2014). Combined treatment with sodium butyrate and PD153035 enhances keratinocyte differentiation. Exp. Dermatol..

[87] Wang Y., Kao M.S., Yu J., Huang S., Marito S., Gallo R.L., Huang C.M. (2016). A precision microbiome approach using sucrose for selective augmentation of *Staphylococcus epidermidis* fermentation against *Propionibacterium acnes*. Int. J. Mol. Sci..

[88] Carneiro L.A., Travassos L.H. (2013). The Interplay between NLRs and Autophagy in Immunity and Inflammation. Front. Immunol..

[89] Platnich J.M., Muruve D.A. (2019). NOD-like receptors and inflammasomes: A review of their canonical and non-canonical signaling pathways. Arch. Biochem. Biophys..

[90] Negishi H., Yanai H., Nakajima A., Koshiba R., Atarashi K., Matsuda A., Matsuki K., Miki S., Doi T., Aderem A. (2012). Cross-interference of RLR and TLR signaling pathways modulates antibacterial T cell responses. Nat. Immunol..

[91] Suez J., Zmora N., Elinav E. (2020). Probiotics in the next-generation sequencing era. Gut Microbes.

[92] Evans S.R. (2010). Clinical trial structures. J. Exp. Stroke Transl. Med..

