# Supplementary material for: Evaluating the Adjuvant Therapeutic Effects of Probiotic Strains Lactococcus cremoris and Lacticaseibacillus paracasei on Canine Atopic Dermatitis and Their Impact on the Gut and Skin Microbiome
Source: Animals (Basel). 2025 Oct 24;15(21):3098. doi: 10.3390/ani15213098 (PMC12609542; doi:10.3390/ani15213098)
Supplement: Supplementary file 1 [file animals-15-03098-s001.zip › animals-3864106-supplementary.pdf]

Supplementary Table S1. Demographic characteristics of recruitment dogs

**Cases No**	**Breed**	**Age (years)**	**Life stage**	**Weight (kg)**	**Gender**	**Treatments**
D1	Labrador Retriever	> 8	Senior	32.0	female	–**
D2	Chihuahua	> 8	Senior	2.7	female	–**
D3	Bichon	1–3	Young	5.5	male	–**
D4	Poodle	> 8	Senior	3.4	male	glucocorticoid
D6	Mixed	3–7	Adult	13.7	male	–**
D7	Schnauzer	3–7	Adult	7.3	female	–**
D8	Mixed	3–7	Adult	16.7	male	glucocorticoid
D9	Shiba Inu	1–3	Young	13.4	female	–**
Summary				10.4 (2.7–32.0)*	Male: 4 (50%); Female: 4 (50%)	

*The results were displayed as median (range).

**Without treatment

Supplementary Table S2. CADESI scores and PVAS scores of recruitment dogs before LCP treatment

Cases No	CADESI scores	PVAS scores	Severity
D1	54	8	Moderate
D2	12	5	Mild
D3	1	7	Normal or Remission
D4	57	6	Moderate
D6	11	4	Mild
D7	27	8	Mild
D8	108	4	Severe
D9	8	6	Normal or Remission

CADESI, Canine Atopic Dermatitis Extent and Severity Index; PVAS, Pruritus Visual Analogue Scale.

Supplementary Table S3. Estimated marginal means, confidence intervals, and overall time effects for CADESI and PVAS scores in dogs with atopic dermatitis (AD) before, during, and after LCP treatment

Indicators	Before LCP treatment	In LCP treatment	After LCP treatment	*P* value
	Day 0	Day 30	Day 60	Overall
CADESI scores	34.8 (4.4–65.1)	23.0 (4.6–41.4)	21.9 (3.4–40.3)	0.68
PVAS scores	6.7 (4.4–9.0)	5.2 (2.6–7.7)	5.7 (3.1–8.2)	0.32

Data were presented as estimated marginal means with 95% confidence intervals. *P*-values for overall time effects were derived from linear mixed-effects model (LMM) analyses using Type III test of fixed effects. Pairwise comparisons between time points were performed with Bonferroni correction; all *P*-values were > 0.05 and were not shown here.

CADESI, Canine Atopic Dermatitis Extent and Severity Index; PVAS, Pruritus Visual Analogue Scale.

Supplementary Table S4. Relative abundance of dominant fecal bacterial phyla before and after 60 days of LCP treatment in dogs with atopic dermatitis

Bacterial phyla	Relative abundance		Directional Mean Change (Day 60 vs. Day 0)		
	Day 0	Day 60		*p* value	q value
Firmicutes	95.836 (78.992–100.000)	96.268 (78.095–100.000)	Increase	>0.99	>0.99
Proteobacteria	0.028 (0.000–20.928)	0.913 (0.000–19.560)	Increase	0.81	>0.99
Actinobacteria	0.741 (0.000–14.392)	1.178 (0.000–4.926)	Decrease	0.63	>0.99
Bacteroidetes	0.011 (0.000–1.088)	0.100 (0.000–2.345)	Increase	0.63	>0.99
Fusobacteria	0.000 (0.000–0.409)	0.000 (0.000–0.000)	Decrease	0.50	>0.99

Relative abundance values were presented as median (range). Directional change indicated the qualitative shift in median abundance between Day 0 and Day 60. *p*-values were calculated using the Wilcoxon signed-rank test. Q-values represent FDR-adjusted p-values. Results were based on paired samples from six dogs.

Supplementary Table S5. Relative abundance of dominant fecal bacterial families before and after 60 days of LCP treatment in dogs with atopic dermatitis

Bacterial families	Relative abundance		Directional Mean Change (Day 60 vs. Day 0)		
	Day 0	Day 60		*p* value	q value
Lachnospiraceae	55.651 (30.709–87.169)	73.923 (16.944–96.594)	Increase	>0.99	>0.99
Peptostreptococcaceae	7.860 (0.055–39.473)	6.040 (0.286–63.410)	Decrease	0.84	0.95
Clostridiaceae	0.816 (0.129–65.358)	0.782 (0.072–8.725)	Decrease	0.69	0.87
Streptococcaceae	0.089 (0.000–2.482)	0.124 (0.000–42.499)	Increase	0.30	0.63
Erysipelotrichaceae	4.403 (1.013–10.895)	2.610 (0.195–7.650)	Decrease	0.03	0.32
Enterobacteriaceae	0.022 (0.000–20.928)	0.913 (0.000–19.560)	Increase	0.63	0.87
Coriobacteriaceae	0.321 (0.000–14.364)	1.139 (0.000–4.914)	Increase	0.63	0.87
Enterococcaceae	0.394 (0.000–12.222)	0.162 (0.000–2.495)	Decrease	0.25	0.63
Lactobacillaceae	0.066 (0.000–6.639)	0.013 (0.000–0.257)	Decrease	0.30	0.60
Oscillospiraceae	0.000 (0.000–1.142)	0.018 (0.000–3.791)	Increase	0.25	0.63

Relative abundance values were presented as median (range). Directional change indicated the qualitative shift in median abundance between Day 0 and Day 60. *p*-values were calculated using the Wilcoxon signed-rank test. Q-values represent FDR-adjusted p-values. Results were based on paired samples from six dogs.

Supplementary Table S6. Relative abundance of dominant fecal bacterial genera before and after 60 days of LCP treatment in dogs with atopic dermatitis

Bacterial genera	Relative abundance		Directional Mean Change (Day 60 vs. Day 0)		
	Day 0	Day 60		*p* value	q value
Blautia	31.785 (11.699–73.278)	54.221 (2.558–91.440)	Increase	>0.99	>0.99
Peptacetobacter	0.000 (0.000–39.177)	0.000 (0.000–63.082)	Increase	>0.99	>0.99
Faecalimonas	8.426 (3.194–35.899)	4.263 (0.323–21.434)	Decrease	0.31	0.63
Sarcina	0.000 (0.000–63.898)	0.000 (0.000–0.000)	Decrease	>0.99	>0.99
Mediterraneibacter	6.711 (2.564–8.095)	5.387 (0.839–8.737)	Decrease	0.31	0.63
Lactococcus	0.006 (0.000–2.299)	0.088 (0.000–42.060)	Increase	0.16	0.52
Shigella	0.000 (0.000–17.413)	0.913 (0.000–12.693)	Increase	0.88	>0.99
Erysipelatoclostridium	2.405 (0.475–10.895)	1.804 (0.063–4.774)	Decrease	0.16	0.52
Collinsella	0.321 (0.000–14.364)	1.139 (0.000–4.914)	Decrease	0.63	>0.99
Romboutsia	0.508 (0.047–12.931)	0.150 (0.025–9.494)	Decrease	0.09	0.52

Relative abundance values were presented as median (range). Directional change indicated the qualitative shift in median abundance between Day 0 and Day 60. *p*-values were calculated using the Wilcoxon signed-rank test. Q-values represent FDR-adjusted p-values. Results were based on paired samples from six dogs.

Supplementary Table S7. Relative abundance of dominant fecal bacterial species before and after 60 days of LCP treatment in dogs with atopic dermatitis

Bacterial species	Relative abundance		Directional Mean Change (Day 60 vs. Day 0)		
	Day 0	Day 60		*p* value	q value
Peptacetobacter hiranonis	0.000 (0.000–39.177)	0.000 (0.000–63.082)	Increase	>0.99	>0.99
Blautia caecimuris	1.396 (0.000–42.823)	0.317 (0.000–45.486)	Decrease	>0.99	>0.99
Blautia schinkii	3.324 (0.000–33.953)	3.446 (0.000–46.247)	Increase	0.56	0.95
Faecalimonas umbilicata	8.426 (3.194–35.899)	4.263 (0.323–21.434)	Decrease	0.31	0.95
Blautia coccoides	1.043 (0.000–19.270)	1.187 (0.000–34.183)	Increase	0.84	>0.99
Mediterraneibacter [Ruminococcus] gnavus ATCC 2914*9*	4.841 (2.564–8.095)	4.617 (0.736–8.116)	Decrease	0.44	0.95
Blautia glucerasea	0.981 (0.243–9.617)	5.613 (0.103–13.220)	Increase	0.56	0.95
Blautia argi	0.903 (0.000–20.454)	0.038 (0.000–19.961)	Decrease	0.88	>0.99
Lactococcus cremoris	0.000 (0.000–2.299)	0.088 (0.000–42.060)	Increase	0.13	0.95
Blautia hansenii DSM 20583	0.373 (0.000–13.872)	1.300 (0.000–13.219)	Decrease	0.44	0.95

Relative abundance values were presented as median (range). Directional change indicated the qualitative shift in median abundance between Day 0 and Day 60. *p*-values were calculated using the Wilcoxon signed-rank test. Q-values represent FDR-adjusted p-values. Results were based on paired samples from six dogs.

Supplementary Table S8. Relative abundance of carbohydrate metabolism and immune system associated pathways in the fecal microbiome of dogs with atopic dermatitis before and after 60 days of LCP treatment

Functional predictions	Relative abundance		Directional Mean Change (Day 60 vs. Day 0)		
	Day 0	Day 60		*p* value	q value
Carbohydrate metabolism					
Glycolysis / Gluconeogenesis	1.299 (1.089–1.796)	1.374 (1.034–1.943)	Increase	>0.99	>0.99
Citrate cycle (TCA cycle)	0.410 (0.401–0.449)	0.430 (0.352–0.542)	Increase	0.56	>0.99
Pentose phosphate pathway	0.789 (0.729–0.931)	0.726 (0.594–0.876)	Decrease	0.03	0.44
Pentose and glucuronate interconversions	0.387 (0.268–0.528)	0.361 (0.258–0.440)	Decrease	0.69	>0.99
Fructose and mannose metabolism	0.741 (0.618–2.248)	0.785 (0.555–1.465)	Decrease	>0.99	>0.99
Galactose metabolism	0.973 (0.827–1.079)	0.970 (0.779–1.056)	Decrease	0.31	0.88
Ascorbate and aldarate metabolism	0.080 (0.061–0.368)	0.098 (0.036–0.258)	Increase	0.84	>0.99
Starch and sucrose metabolism	1.608 (1.394–1.885)	1.502 (1.180–2.615)	Increase	>0.99	>0.99
Amino sugar and nucleotide sugar metabolism	1.704 (1.548–2.408)	1.647 (1.492–2.483)	Decrease	0.69	>0.99
Inositol phosphate metabolism	0.134 (0.127–0.160)	0.131 (0.099–0.153)	Decrease	0.56	>0.99
Pyruvate metabolism	1.069 (1.045–1.111)	1.088 (1.041–1.170)	Increase	0.09	0.44
Propanoate metabolism	0.580 (0.522–0.781)	0.714 (0.513–0.823)	Increase	0.06	0.44
Butanoate metabolism	0.369 (0.311–0.774)	0.454 (0.331–0.725)	Increase	0.22	0.77
C5-Branched dibasic acid metabolism	0.190 (0.159–0.217)	0.211 (0.097–0.233)	Decrease	>0.99	>0.99
Immune system					
Antigen processing and presentation	0.021 (0.013–0.024)	0.022 (0.008–0.026)	Decrease	>0.99	>0.99

NOD-like receptor signaling pathway	0.075 (0.055–0.097)	0.071 (0.049–0.110)	Decrease	0.56	>0.99
RIG-I-like receptor signaling pathway	0.002 (0.000–0.028)	0.003 (0.000–0.032)	Increase	0.44	>0.99
IL-17 signaling pathway	0.021 (0.013–0.024)	0.022 (0.008–0.026)	Decrease	>0.9	>0.99
Th17 cell differentiation	0.021 (0.013–0.024)	0.022 (0.008–0.026)	Decrease	>0.99	>0.99

Relative abundance values were presented as median (range). Directional change indicated the qualitative shift in median abundance between Day 0 and Day 60. *p*-values were calculated using the Wilcoxon signed-rank test. Q-values represent FDR-adjusted p-values. Results were based on paired samples from six dogs.

Supplementary Table S9. Relative abundance of dominant skin bacterial phyla before and after 60 days of LCP treatment in dogs with atopic dermatitis

Bacterial phyla	Relative abundance		Directional Mean Change (Day 60 vs. Day 0)		
	Day 0	Day 60		*p* value	q value
Firmicutes	55.830 (16.121–96.212)	72.797 (7.878–98.334)	Increase	0.69	0.78
Proteobacteria	19.500 (1.645–50.855)	5.557 (0.494–50.410)	Decrease	0.44	0.66
Actinobacteria	5.092 (1.842–23.644)	9.953 (0.710–79.274)	Increase	0.44	0.66
Bacteroidetes	3.355 (0.217–22.653)	1.739 (0.089–7.007)	Decrease	0.56	0.73
Fusobacteria	0.639 (0.000–4.608)	0.145 (0.000–1.291)	Decrease	0.44	0.66
Cyanobacteria	0.998 (0.052–2.029)	0.114 (0.000–0.412)	Decrease	0.16	0.47
Deinococcus-Thermus	0.778 (0.000–1.278)	0.100 (0.000–0.887)	Decrease	0.16	0.47
Acidobacteria	0.188 (0.000–1.062)	0.000 (0.000–2.235)	Increase	0.88	0.88
Planctomycetes	0.118 (0.000–0.599)	0.000 (0.000–0.198)	Decrease	0.06	0.47

Relative abundance values were presented as median (range). Directional change indicated the qualitative shift in median abundance between Day 0 and Day 60. *p*-values were calculated using the Wilcoxon signed-rank test. Q-values represent FDR-adjusted p-values. Results were based on paired samples from six dogs.

Supplementary Table S10. Relative abundance of dominant skin bacterial families before and after 60 days of LCP treatment in dogs with atopic dermatitis

Bacterial families	Relative abundance		Directional Mean Change (Day 60 vs. Day 0)		
	Day 0	Day 60		*p* value	q value
Staphylococcaceae	27.440 (0.687–87.561	49.263 (0.170–96.482)	Increase	0.22	0.44
Propionibacteriaceae	1.352 (0.323–9.004)	4.677 (0.154–72.525)	Increase	0.22	0.44
Streptococcaceae	2.802 (0.988–34.986)	1.987 (1.291–33.074)	Decrease	0.56	0.79
Moraxellaceae	0.737 (0.093–2.385)	0.614 (0.000–20.378)	Increase	0.84	0.85
Neisseriaceae	0.803 (0.000–7.285)	0.881 (0.000–14.531)	Increase	0.63	0.79
Lactobacillaceae	2.514 (0.186–4.223)	0.991 (0.103–2.445)	Decrease	0.06	0.32
Porphyromonadaceae	0.529 (0.000–16.034)	0.851 (0.000–2.005)	Decrease	0.84	0.85
Burkholderiaceae	0.157 (0.021–15.420)	0.233 (0.000–2.805)	Decrease	0.56	0.79
Sphingomonadaceae	1.648 (0.093–10.054)	0.102 (0.000–1.227)	Decrease	0.09	0.32
Actinomycetaceae	0.333 (0.135–14.121)	0.026 (0.000–4.767)	Decrease	0.03	0.32

Relative abundance values were presented as median (range). Directional change indicated the qualitative shift in median abundance between Day 0 and Day 60. *p*-values were calculated using the Wilcoxon signed-rank test. Q-values represent FDR-adjusted p-values. Results were based on paired samples from six dogs.

Supplementary Table S11. Relative abundance of dominant skin bacterial genera before and after 60 days of LCP treatment in dogs with atopic dermatitis

Bacterial genera	Relative abundance		Directional Mean Change (Day 60 vs. Day 0)		
	Day 0	Day 60		*p* value	q value
Staphylococcus	27.440 (0.687–87.561)	49.221 (0.170–96.482)	Increase	0.22	0.51
Cutibacterium	1.159 (0.000–9.004)	4.488 (0.123–72.525)	Increase	0.22	0.51
Streptococcus	2.786 (0.988–34.986)	1.941 (1.265–33.074)	Decrease	0.56	0.95
Porphyromonas	0.529 (0.000–16.034)	0.851 (0.000–2.005)	Decrease	0.84	0.95
Conchiformibius	0.246 (0.000–6.597)	0.290 (0.000–14.531)	Increase	0.81	0.95
Acinetobacter	0.126 (0.000–0.518)	0.000 (0.000–17.513)	Increase	0.81	0.95
Lautropia	0.010 (0.000–15.371)	0.213 (0.000–2.645)	Decrease	>0.99	>0.99
Arcanobacterium	0.067 (0.000–14.121)	0.026 (0.000–4.149)	Decrease	0.25	0.51
Sphingomonas	1.648 (0.041–7.892)	0.020 (0.000–1.227)	Decrease	0.09	0.51
Lactobacillus	1.529 (0.000–2.961)	0.375 (0.021–1.566)	Decrease	0.22	0.51

Relative abundance values were presented as median (range). Directional change indicated the qualitative shift in median abundance between Day 0 and Day 60. *p*-values were calculated using the Wilcoxon signed-rank test. Q-values represent FDR-adjusted p-values. Results were based on paired samples from six dogs.

Supplementary Table S12. Relative abundance of dominant skin bacterial species before and after 60 days of LCP treatment in dogs with atopic dermatitis

Bacterial species	Relative abundance		Directional Mean Change (Day 60 vs. Day 0)		
	Day 0	Day 60		*p* value	q value
Staphylococcus pseudintermedius	20.900 (0.000–87.002)	10.717 (0.000–81.297)	Decrease	>0.99	>0.99
Cutibacterium acnes	1.159 (0.000–9.004)	4.473 (0.123–72.525)	Increase	0.22	0.84
Staphylococcus coagulans	0.338 (0.000–7.845)	3.738 (0.000–88.530)	Increase	0.25	0.84
Streptococcus canis	0.000 (0.000–34.669)	0.248 (0.000–32.839)	Decrease	>0.99	>0.99
Staphylococcus capitis	0.113 (0.000–9.217)	0.539 (0.000–11.726)	Increase	0.63	>0.99
Conchiformibius steedae	0.246 (0.000–6.597)	0.290 (0.000–14.531)	Increase	0.81	>0.99
Lautropia mirabilis	0.010 (0.000–15.371)	0.213 (0.000–2.645)	Decrease	>0.99	>0.99
Arcanobacterium wilhelmae	0.067 (0.000–(14.121)	0.026 (0.000–4.149)	Decrease	0.25	0.84
Acinetobacter indicus CIP 110367	0.000 (0.000–0.000)	0.000 (0.000–15.127)	Increase	>0.99	>0.99
Porphyromonas cangingivalis	0.449 (0.000–10.242)	0.000 (0.000–0.917)	Decrease	0.44	>0.99

Relative abundance values were presented as median (range). Directional change indicated the qualitative shift in median abundance between Day 0 and Day 60. *p*-values were calculated using the Wilcoxon signed-rank test. Q-values represent FDR-adjusted p-values. Results were based on paired samples from six dogs.

Supplementary Table S13. Relative abundance of immune system and bacterial infectious diseases associated pathways in the skin microbiome of dogs with atopic dermatitis before and after 60 days of LCP treatment

Functional predictions	Relative abundance		Directional Mean Change (Day 60 vs. Day 0)		
	Day 0	Day 60		*p* value	q value
Immune system					
Antigen processing and presentation	0.005 (0.002–0.010)	0.003 (0.000–0.009)	Decrease	0.44	0.52
NOD-like receptor signaling pathway	0.005 (0.033–0.087)	0.045 (0.030–0.067)	Decrease	0.44	0.52
RIG-I-like receptor signaling pathway	0.020 (0.007–0.032)	0.030 (0.021–0.040)	Increase	0.09	0.52
C-type lectin receptor signaling pathway	0.000 (0.000–0.000)	0.000 (0.000–0.000)	Increase	>0.99	>0.99
IL-17 signaling pathway	0.005 (0.002–0.010)	0.003 (0.000–0.009)	Decrease	0.44	0.52
Th17 cell differentiation	0.005 (0.002–0.010)	0.003 (0.000–0.009)	Decrease	0.44	0.52
Fc gamma R-mediated phagocytosis	0.001 (0.000–0.005)	0.000 (0.000–0.002)	Decrease	0.44	0.52
Bacterial infectious diseases					
Bacterial invasion of epithelial cells	0.047 (0.002–0.083)	0.066 (0.000–0.090)	Increase	0.44	0.59
Vibrio cholerae infection	0.003 (0.000–0.007)	0.001 (0.000–0.003)	Decrease	0.22	0.44
Epithelial cell signaling in Helicobacter pylori infection	0.050 (0.035–0.068)	0.052 (0.012–0.071)	Decrease	0.56	0.65
Pathogenic Escherichia coli infection	0.000 (0.000–0.000)	0.000 (0.000–0.000)	Decrease	0.13	0.34
Shigellosis	0.000 (0.000–0.000)	0.000 (0.000–0.000)	Decrease	0.06	0.34
Salmonella infection	0.036 (0.020–0.091)	0.025 (0.006–0.028)	Decrease	0.09	0.34
Staphylococcus aureus infection	0.222 (0.023–0.472)	0.318 (0.011–0.511)	Increase	0.31	0.51

Tuberculosis	0.084 (0.074–0.092)	0.084 (0.072–0.097)	Unchange	>0.99	>0.99

Relative abundance values were presented as median (range). Directional change indicated the qualitative shift in median abundance between Day 0 and Day 60. *p*-values were calculated using the Wilcoxon signed-rank test. Q-values represent FDR-adjusted p-values. Results were based on paired samples from six dogs.

Supplementary Table S14. Spearman correlation coefficients (r), 95% confidence intervals (CI), and *p*-values between selected bacterial taxa and immunological indicators (IgE, IL-4, and IFN-γ)

	CADESI		IgE		IL4		IFNr	
	r (95% CI)	*p* value	*r* (95% CI)	*p* value	*r* (95% CI)	*p* value	*r* (95% CI)	*p* value
Erysipelotrichaceae	0.57 (-0.03 to 0.87)	0.06	0.22 (-0.42 to 0.72)	0.49	0.20 (-0.44 to 0.71)	0.73	0.10 (-0.52 to 0.65)	0.76
Romboutsia	0.16 (-0.47 to 0.68)	0.62	0.46 (-0.17 to 0.82)	0.13	-0.40 (-0.80 to 0.25)	0.04	0.11 (-0.51 to 0.66)	0.73
Enterobacteriaceae	0.11 (-0.51 to 0.66)	0.73	0.35 (-0.29 to 0.78)	0.26	0.13 (-0.49 to 0.67)	0.80	-0.27 (-0.74 to 0.38)	0.40
Escherichia/Shigella spp.	0.26 (-0.38 to 0.74)	0.41	0.14 (-0.49 to 0.67)	0.67	-0.07(-0.63 to 0.54)	0.80	-0.17 (-0.69 to 0.46)	0.60
Shigella flexneri	0.29 (-0.36 to 0.75)	0.35	0.21 (-0.43 to 0.71)	0.50	-0.22 (-0.72 to 0.42)	0.66	-0.09 (-0.65 to 0.52)	0.77

Spearman correlation analysis between microbial taxa and immunological markers (serum IgE, IL-4, and IFN-γ). The table included correlation coefficients (r), 95% confidence intervals, p-values, and significance interpretations (α = 0.05). These results reflected statistical uncertainty and support interpretation of correlation strength between microbial taxa and host immune indicators.

(A)

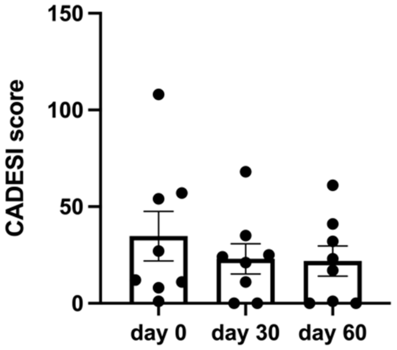

(B)

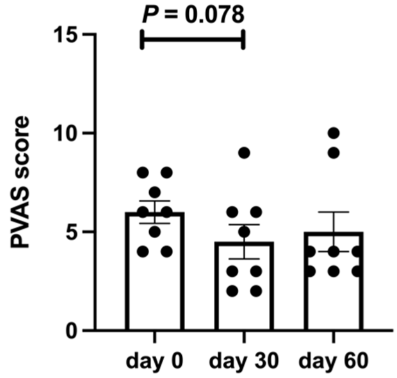

Supplementary Figure S1. Clinical assessment of atopic dermatitis severity over time following LCP treatment. CADESI (A) and PVAS (B) scores were evaluated at baseline (Day 0), Day 30, and Day 60 after LCP treatment. CADESI scores were analyzed using a paired t-test, and PVAS scores were analyzed using the Wilcoxon signed-rank test. Data were presented as mean ± SEM (n = 8).

(A)

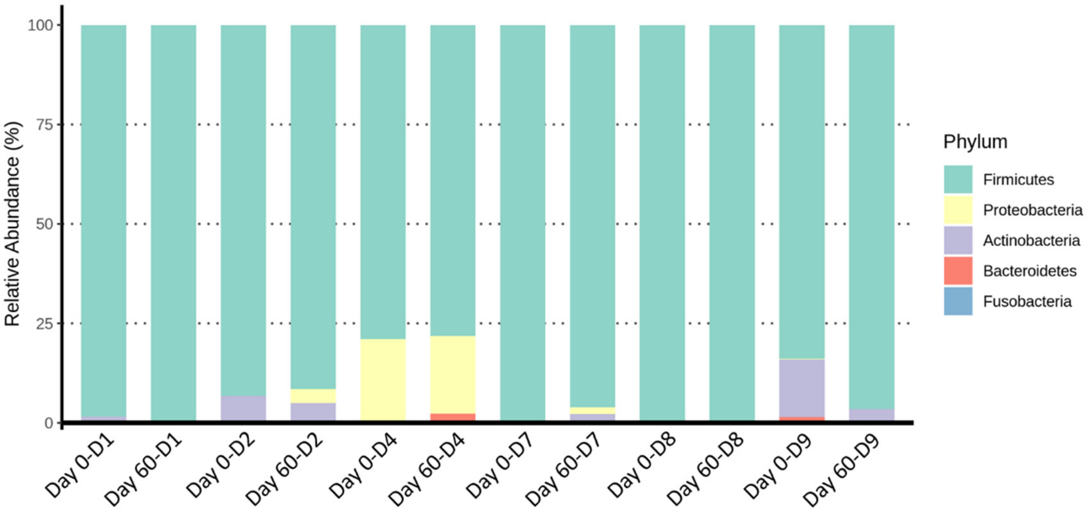

Relative Abundance (%)
100
75
50
25
0
Day 0-D1
Day 60-D1
Day 0-D2
Day 60-D2
Day 0-D4
Day 60-D4
Day 0-D7
Day 60-D7
Day 0-D8
Day 60-D8
Day 0-D9
Day 60-D9
Phylum
Firmicutes
Proteobacteria
Actinobacteria
Bacteroidetes
Fusobacteria

(B)

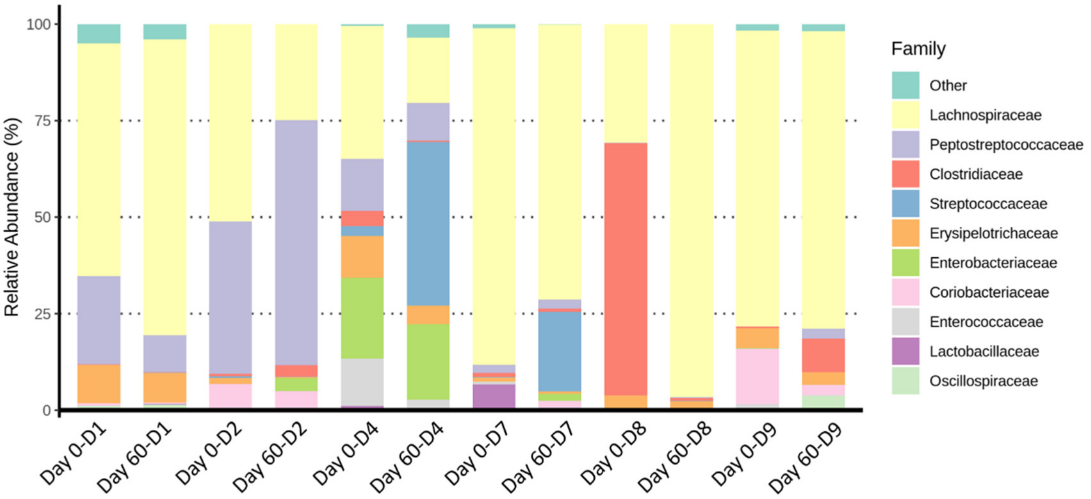

Relative Abundance (%)
100
75
50
25
0
Day 0-D1
Day 60-D1
Day 0-D2
Day 60-D2
Day 0-D4
Day 60-D4
Day 0-D7
Day 60-D7
Day 0-D8
Day 60-D8
Day 0-D9
Day 60-D9
Family
Other
Lachnospiraceae
Peptostreptococcaceae
Clostridiaceae
Streptococcaceae
Erysipelotrichaceae
Enterobacteriaceae
Coriobacteriaceae
Enterococcaceae
Lactobacillaceae
Oscillospiraceae

(C)

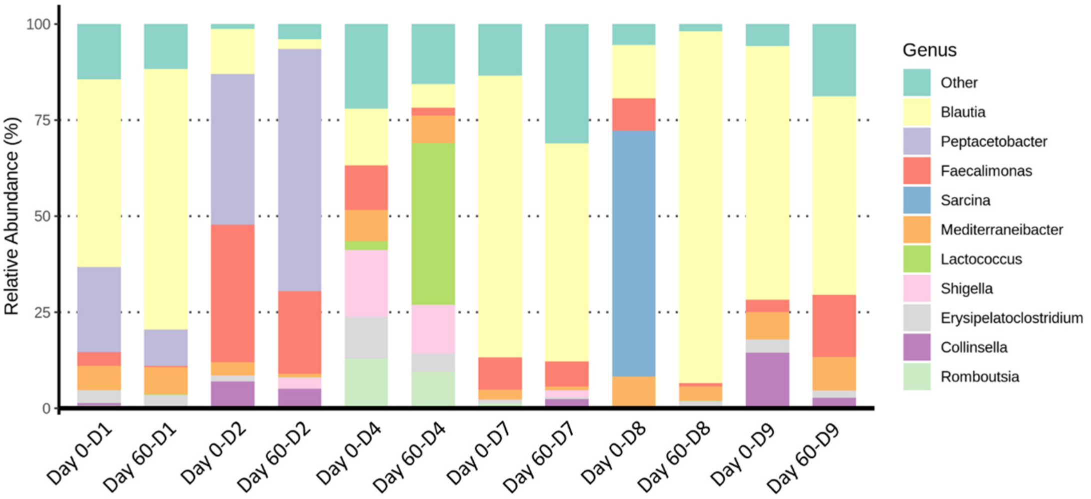

Relative Abundance (%)
100
75
50
25
0
Day 0-D1
Day 60-D1
Day 0-D2
Day 60-D2
Day 0-D4
Day 60-D4
Day 0-D7
Day 60-D7
Day 0-D8
Day 60-D8
Day 0-D9
Day 60-D9
Genus
Other
Blautia
Peptacetobacter
Faecalimonas
Sarcina
Mediterraneibacter
Lactococcus
Shigella
Erysipelatoclostridium
Collinsella
Romboutsia

(D)

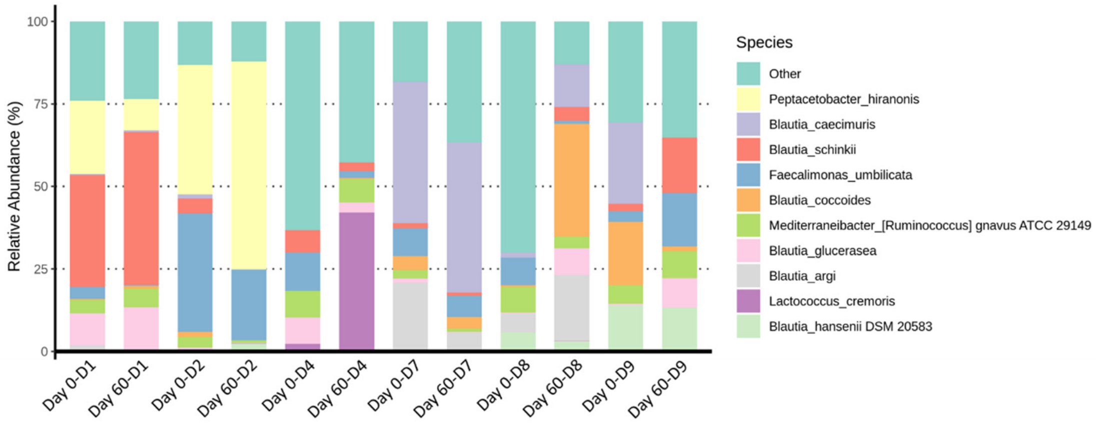

Supplementary Figure S2. Stacked bar plots showing the relative abundances of the dominant fecal bacterial taxa in individual dogs with atopic dermatitis at (A) phylum, (B) family, (C) genus, and (D) species levels.

(A)

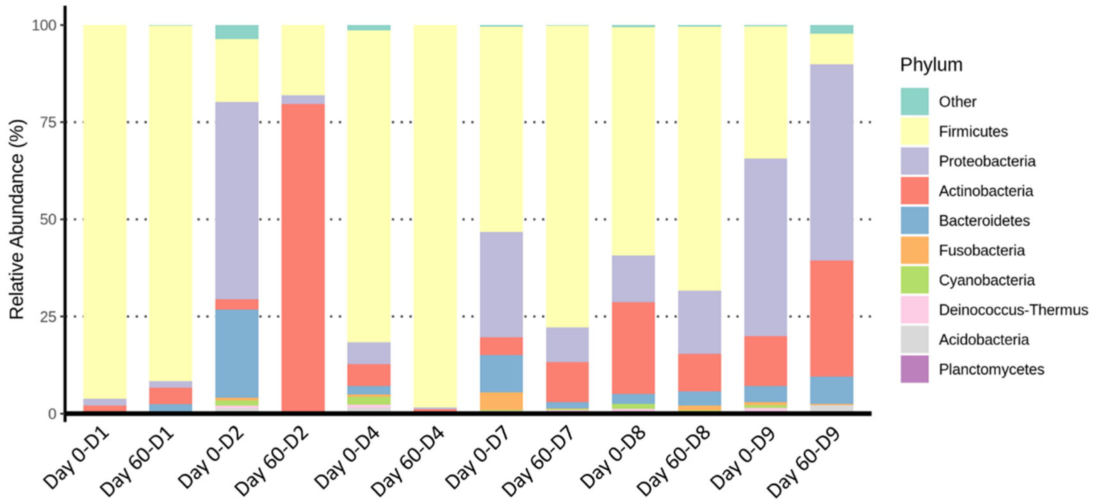

Relative Abundance (%)
100
75
50
25
0
Day 0-D1
Day 60-D1
Day 0-D2
Day 60-D2
Day 0-D4
Day 60-D4
Day 0-D7
Day 60-D7
Day 0-D8
Day 60-D8
Day 0-D9
Day 60-D9
Phylum
Other
Firmicutes
Proteobacteria
Actinobacteria
Bacteroidetes
Fusobacteria
Cyanobacteria
Deinococcus-Thermus
Acidobacteria
Planctomycetes

(B)

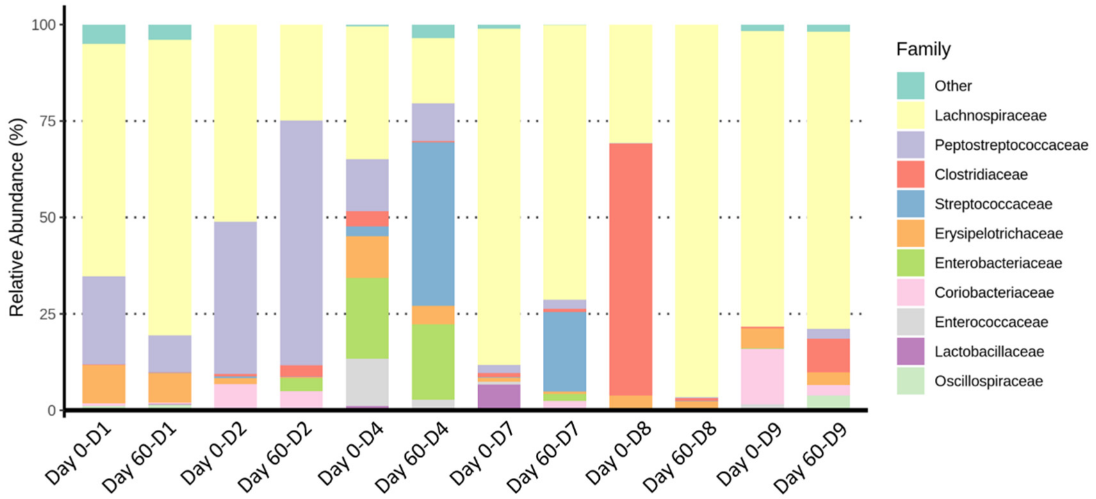

Relative Abundance (%)
100
75
50
25
0
Day 0-D1
Day 60-D1
Day 0-D2
Day 60-D2
Day 0-D4
Day 60-D4
Day 0-D7
Day 60-D7
Day 0-D8
Day 60-D8
Day 0-D9
Day 60-D9
Family
Other
Lachnospiraceae
Peptostreptococcaceae
Clostridiaceae
Streptococcaceae
Erysipelotrichaceae
Enterobacteriaceae
Coriobacteriaceae
Enterococcaceae
Lactobacillaceae
Oscillospiraceae

(C)

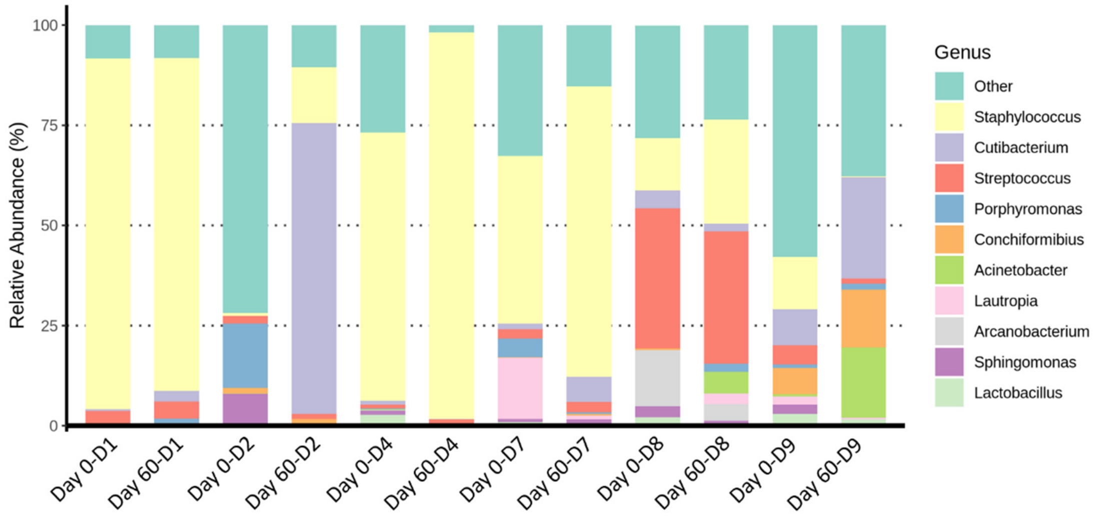

Relative Abundance (%)
100
75
50
25
0
Day 0-D1
Day 60-D1
Day 0-D2
Day 60-D2
Day 0-D4
Day 60-D4
Day 0-D7
Day 60-D7
Day 0-D8
Day 60-D8
Day 0-D9
Day 60-D9
Genus
Other
Staphylococcus
Cutibacterium
Streptococcus
Porphyromonas
Conchiformibius
Acinetobacter
Lautropia
Arcanobacterium
Sphingomonas
Lactobacillus

(D)

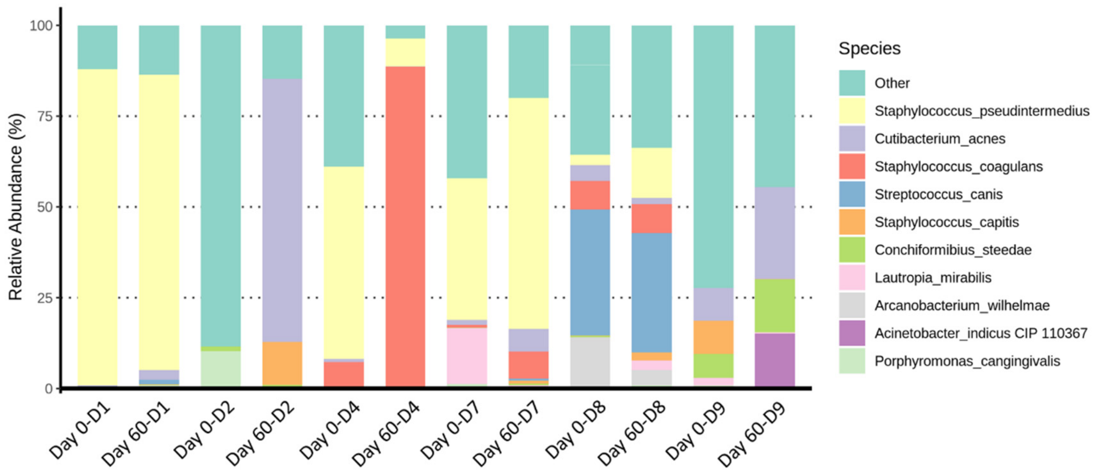

Supplementary Figure S3. Stacked bar plots showing the relative abundances of the dominant skin bacterial taxa in individual dogs with atopic dermatitis at (A) phylum, (B) family, (C) genus, and (D) species levels.
